# A Researcher’s guide to rodent models of Down syndrome: Recent insights and translational perspectives

**DOI:** 10.1016/j.xpro.2026.104717

**Published:** 2026-07-24

**Authors:** Mir A. Raza, Andrew Folz, Daniella B. Victorino, Zhuo Xing, Xu-Qiao Chen, Xinxin Zuo, Leah K. Borden, Marie-Claude Potier, William Mobley, Mara Dierssen, Masaharu Hiratsuka, Yasuhiro Kazuki, Kristy Welshhans, Michelle Maugham-Macan, Antonella Tramutola, Eitan Okun, Roger Reeves, Randall J. Roper, Y. Eugene Yu, Yann Hérault, Victor L.J. Tybulewicz, Elizabeth M.C. Fisher, Aaron Sathyanesan

**Affiliations:** 1 Department of Biology, College of Arts & Sciences, University of Dayton, Dayton, OH 45469, USA; 2 Department of Biology, Indiana University, Indianapolis, IN 46202, USA; 3 Institut du Cerveau - Paris Brain Institute - ICM, CNRS, APHP, Hôpital de La Pitié Salpêtrière, Inserm, Sorbonne Université, Paris, France; 4 The Children’s Guild Foundation Down Syndrome Research Program, Department of Cancer Genetics and Genomics, Roswell Park Comprehensive Cancer Center, Buffalo, NY 14263, USA; 5 Department of Neurosciences, University of California, San Diego, La Jolla, CA 92093, USA; 6 Department of Biology, Massachusetts Institute of Technology, Cambridge, MA 02139, USA; 7 Alana Down Syndrome Center, Massachusetts Institute of Technology, Cambridge, MA 02139, USA; 8 Centre for Genomic Regulation (CRG), The Barcelona Institute of Science and Technology, University Pompeu Fabra, Barcelona, Biomedical Research Networking Center for Rare Diseases (CIBERER), 28036 Barcelona, Spain; 9 Chromosome Engineering Research Center, Tottori University, 86 Nishi-cho, Yonago, Tottori 683-8503, Japan; 10 Department of Biological Sciences, University of South Carolina, Columbia, SC 29208, USA; 11 Carolina Autism and Neurodevelopment (CAN) Research Center, University of South Carolina, Columbia, SC 29208, USA; 12 School of Health, University of the Sunshine Coast, Sippy Downs QLD 4556, Queensland, Australia; 13 Department of Biochemical Sciences ‘A. Rossi-Fanelli’, Sapienza University of Rome, 00185 Rome, Italy; 14 The Mina and Everard Goodman Faculty of Life Sciences, Bar-Ilan University Ramat Gan, Ramat Gan 5290002, Israel; 15 Department of Physiology, Pharmacology and Therapeutics, Johns Hopkins University School of Medicine, Baltimore, MD 21205, USA; 16 Université of Strasbourg, CNRS, INSERM, Institut de Génétique et de Biologie Moléculaire et Cellulaire (IGBMC), UMR7104 U1258, 1 Rue Laurent Fries, 67404 Illkirch, France; 17 Université of Strasbourg, CNRS, INSERM, PHEN-ICS, UAR2062, US66, Illkirch, France; 18 CNRS, CELPHEDIA Core, UAR2052, US66, Villejuif, France; 19 The Francis Crick Institute, 1 Midland Road, London NW1 1AT, UK; 20 Department of Neuromuscular Diseases, Queen Square Institute of Neurology, University College London, London WC1N 3BG, UK; 21 Department of Electrical & Computer Engineering, School of Engineering, University of Dayton, Dayton, OH 45469, USA

**Keywords:** Developmental biology, Neuroscience, Behavior, NMGN Focused Collection

## Abstract

Rodent models of Down syndrome (DS) have been transformative in identifying basic mechanisms underlying the effects of Trisomy 21 at the molecular, cellular, physiological, and neurobehavioral levels. Each model, with its unique genomic architecture, has advanced our understanding of the complex multisystem etiology of DS. The availability of multiple models necessitates the challenge of selecting appropriate models to address a particular scientific question, experimental design, and translational relevance. This primer guides the reader through the various rodent models of DS and the genomic and phenotypic effects they recapitulate. We also provide recommendations and strategies for using DS rodent models to enable effective and robust forward and reverse translational approaches.

## Graphical abstract

**Figure:**
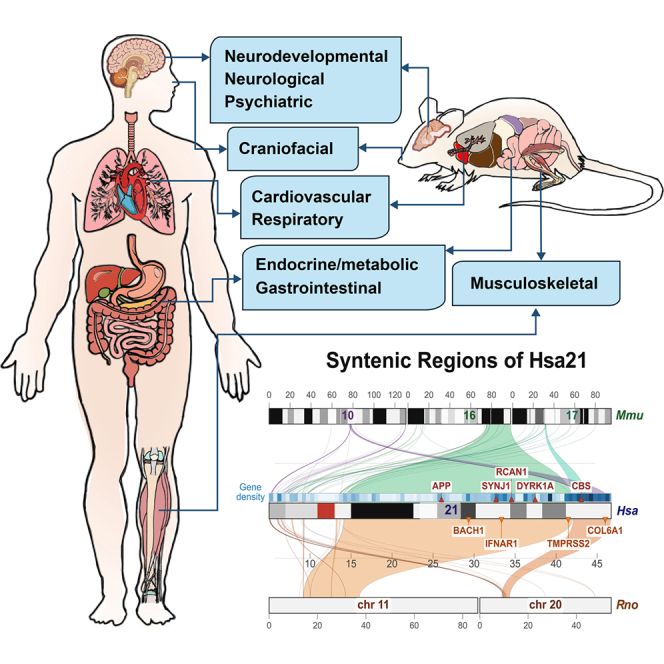

## Introduction

**Figure 1:**
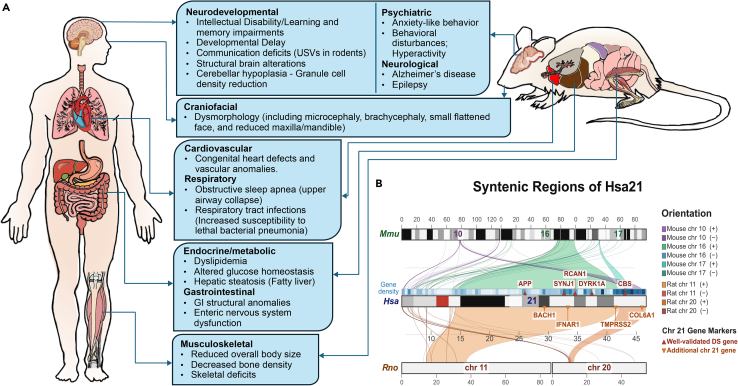
Rodent models of Down syndrome recapitulate major human phenotypes (A) Systems-level phenotypes observed in humans with Down syndrome (DS) that are captured across rodent models of DS. (B) Syntenic regions of Human chromosome 21 (Hsa21) with the mouse (Mus musculus, GRCm39, top) and the rat (rattus norvegicus, GRCr8, bottom). Hsa21 spans the euchromatic region from 5.01 to 46.71 Mb (GRCh38/hg38). Curved ribbons represent top-level net alignment blocks, with colors indicating the orientation: forward strand alignments (+) are shown in lighter shades, while reverse strand alignments (−) are shown in darker shades. In mouse, Hsa21 orthologs are distributed across three chromosomes: Mmu10 (purple), Mmu16 (green/blue), and Mmu17 (teal). In the rat, orthologs map primarily to Rno11 (orange) and Rno20 (dark orange). The gene density track (blue gradient bar above Hsa21) represents the distribution of RefSeq genes across the chromosome in 500 kb bins, with darker blue indicating higher gene density. Markers highlight a few key DS-associated genes and other functionally important Hsa21 genes: *APP* (amyloid precursor protein), *DYRK1A* (dual-specificity tyrosine phosphorylation-regulated kinase 1A), *RCAN1* (regulator of calcineurin 1), *CBS* (cystathionine beta-synthase), *SYNJ1* (synaptojanin 1), *BACH1* (BTB domain and CNC homolog 1), *IFNAR1* (interferon alpha/beta receptor 1), *COL6A1* (collagen type VI alpha 1 chain), and *TMPRSS2* (transmembrane serine protease 2). Genomic coordinates are shown in megabases (Mb) along each chromosome axis. The dashed horizontal lines separate the three species comparisons. Synteny data were obtained from UCSC Genome Browser chain/net alignments, which represent best-in-genome pairwise alignments filtered to remove lower-scoring overlaps. Script used to generate syntenic regions of Hsa21 with the mouse and rat genome in Figure 1B has been uploaded to Github: Github repository.

Down syndrome (DS) is caused by the partial or complete trisomy of human chromosome 21 (Hsa21). This leads to a myriad of phenotypes affecting the genome, proteome, cell physiology, organ systems, and neurobehavioral repertoire of persons with DS^1^^,^^2^ (Figure 1A). A major challenge in DS research lies in defining the pathophysiological consequences of an increased number of protein-coding and non-coding genes on Hsa21, their effects on genome-wide gene expression, and how these changes contribute to clinical manifestations.^1^^,^^3^ Studies using human post-mortem brain tissue and advanced *in vitro* systems have provided valuable insights into the molecular and cellular complexity of DS.^4^^,^^5^^,^^6^^,^^7^^,^^8^^,^^9^^,^^10^^,^^11^ However, these platforms have inherent limitations, particularly in their ability to model biology across tissue-types and organ-systems.^12^ Importantly, these platforms cannot model the atypical neurobehavior associated with complex developmental brain conditions.^13^ Thus, understanding the complexity of pathophysiological processes in DS, and parsing multifactorial and long-term responses to therapeutic interventions, requires a whole-organism approach.^14^

Humans, mice, and rats diverged from a common ancestor around 80 million years ago.^15^ This is a mere blink of evolutionary time, and so our genes and biochemical pathways are largely conserved, hence the power of working with rodent models of human disease, despite the obvious differences in size, lifespan, and some physiology. At the genetic level, Hsa21 has three regions of homology in the mouse genome, on mouse chromosomes 10, 16 and 17 (Mmu10, 16, 17) and two regions of homology in the rat genome on rat chromosomes 11 and 20 (Rnor11, 20) (Figure 1B). The presence of Hsa21 homologous genes on different rodent chromosomes has largely dictated how DS is modelled and has led to some technical tours de force of genome engineering. This has enabled significant expansion of the repertoire of DS rodent models, with notable improvements in both face and construct validity^14^^,^^16^^,^^17^ (Table 1). While the newest generation of DS rodent models continue advancing territory on chromosomal coverage, the segregated triplication of singular genes or groups of genes has allowed for systematic study of causal genotype-to-phenotype relationships.^28^ Beyond forward translation (i.e., from the lab to the clinic), DS rodent models could also support reverse translational approaches (i.e., from the clinic back to the lab), whereby insights from clinical trials and human studies may inform the refinement of experimental targets, model design, and outcome measures. Such a cyclic, iterative, and bidirectional process may further improve the predictive validity of preclinical DS research.

**Table 1:** Rodent Models of Down syndrome

Approved name (MGI; mouse *or* rat database)	Lab or common name	Type of DS model	Short description	Genetic background	Chromosomal Position (MGI; mouse *or* human genomic coordinates *or* rat genomic coordinates)	Number of Hsa21 orthologous genes at dosage imbalance (excluding krtap genes)	First publication	Availability
**a. Mouse Models**								

**Segmental Trisomy Models**								
Ts(17<16>)65Dn (001924) https://www.informatics.jax.org/marker/MGI:2178111	Ts65Dn (001924)	Radiation-induced translocation	Trisomy for a marker chromosome containing a segment of *Mrpl39* to the telomere of Mmu16 homologous to Hsa21, translocated to the centromeric end of Mmu17.	B6EiC3Sn	Breakpoint is Chr16:84351351 bp and Chr17:9426822 bp	101	Davisson MT, et al.,1993 https://pubmed.ncbi.nlm.nih.gov/8115398/	https://www.jax.org/strain/001924
Ts(17<16>)65Dn (005252) https://www.informatics.jax.org/marker/MGI:2178111	Ts65Dn (005252)	Congenic made from a radiation-induced translocation	Trisomy for a marker chromosome containing a segment of *Mrpl39* to the telomere of Mmu16 homologous to Hsa21, translocated to the centromeric end of Mmu17. These mice are homozygous for the wild-type allele of *Pde6b* to eliminate possible retinal degeneration and have slightly different phenotypes than Ts65Dn 001924	B6EiC3Sn.BLiA	Breakpoint is Chr16:84351351 bp and Chr17:9426822 bp	101	Costa AC et al., 2010 https://pubmed.ncbi.nlm.nih.gov/19720087/	https://www.jax.org/strain/005252
Ts(17^16^)66Yah https://www.informatics.jax.org/allele/MGI:6459295	Ts66Yah	CRISPR/Cas9 derivative of Ts65Dn	This mouse strain is derived from the Ts65Dn 01924 strain and removes the non-Hsa21 homologous *Pisd-ps2* to *Pde10a* region derived from Mmu17 in the Ts65Dn mouse using CRISPR/Cas9	B6C3B	–	101	Duchon A, et al.^18^https://pubmed.ncbi.nlm.nih.gov/36374158/	https://www.jax.org/strain/036600
**Translocation Models**								
Ts(16C-tel)1Cje https://www.informatics.jax.org/marker/MGI:3623021	Ts1Cje	Translocation	Mice are trisomic for *Sod1* to *Mx1* and 94 genes on Mmu16 that translocated to Mmu12, deleting genes from Mmu12. *Sod1* is not functionally triplicated because the *Sod1* gene in the translocated segment is inactivated by the insertion of the neomycin resistance sequence. These mice are monosomic for 7 genes in Mmu12 (Duchon A, et al 2011 https://pubmed.ncbi.nlm.nih.gov/21953411/	B6EiC3Sn	Translocation breakpoint between positions 119278499 and 119289499, in the *Dnahc11* gene, between exons 35 and 41.	64	Sago H, et al., 1998. https://pubmed.ncbi.nlm.nih.gov/9600952/	https://www.jax.org/strain/004861
Rb(12.Ts17^16^65Dn)2Cje https://www.informatics.jax.org/allele/MGI:3531161	Ts2Cje	Spontaneous Robertsonian translocation	Spontaneous fusion between the small marker chromosome in Ts65Dn mice (carrying *Mrpl39* to the distal telomere of Mmu16) and Mmu12, forming a Robertsonian fusion chromosome	B6EiC3Sn	–	101	Villar AJ, et al., 2005 https://pubmed.ncbi.nlm.nih.gov/15859352/	https://www.jax.org/strain/004850
**Transchromosomic Models**								
Tc(HSA21)1TybEmcf https://www.informatics.jax.org/marker/MGI:3814702	Tc1	Transchromosomic	Contains a freely segregating copy of Hsa21, with ∼75% of Hsa21 in the mouse. Mice are mosaic for the extra chromosome. The copy of Hsa21 has internal deletions and rearrangements (Gribble SM et al., 2013) https://pubmed.ncbi.nlm.nih.gov/23596509/.	B6129S	Human chr21 coordinates (hg19, per Gribble et al., 2013): Tc1-Hsa21 shows multiple rearrangements; major copy-number changes include deletion Chr21: 18734534–19762829 (∼959 kb) and deletion Chr21:33640510–36370035 (∼2.73 Mb); plus a duplicated segment Chr21:28,490,850–48,680,190 (∼20.19 Mb) with additional complex internal CNVs/rearrangements (see Table 1; Figure 1 in Gribble et al., 2013)	227^a^ (Gribble et al., 2013 https://www.ncbi.nlm.nih.gov/pmc/articles/PMC3626651/) aIncludes duplications and anticipated single-copy genes)	O’Doherty A, et al.^19^https://pubmed.ncbi.nlm.nih.gov/16179473/	https://www.jax.org/strain/010801
Tc(HSA21,CAG-EGFP)1Yakazhttps://www.informatics.jax.org/allele/MGI:6386757	TcMAC21	Transchromosomic	Contains freely segregating copy of Hsa21 with 93% of Hsa21 represented in the mouse.	B6D2	bGRCh38/RefSeq NC_000021.9 (HSA21): loxP insertion 13021348–13028858; HSA21q-MAC span (WGS) 13021348–46691226; deletions (WGS): Del1 14893880–15914720; Del2 17843345–23995878; Del3 26848509–28420310; Del4 45393043–46254350.	144^a^ (Kazuki Y, et al. 2020 https://pubmed.ncbi.nlm.nih.gov/32597754/) a581 total genes which include 199 PCGs and 382 nPCGs; number excludes 49 KRTAPs and six human-specific PCGs.	Kazuki Y, et al.^20^https://pubmed.ncbi.nlm.nih.gov/32597754/	https://www.jax.org/strain/035561
**Duplication Models**								
Dp(16Cbr1-Fam3b)1Rhr https://www.informatics.jax.org/allele/MGI:3487283	Ts1Rhr	Duplication	Cre-mediated chromosomal rearrangement on Mmu16 between loxP sites in regions proximal to *Cbr1* and distal to *Fam3b* results in duplication of the genes, excluding *Cbr1* and including *Fam3b*, in the region that is syntenic with the distal part of Hsa21q22. Also produced the reciprocal deletion as Ms1Rhr.	B6.129S6	Chr16:93404725-97306136	28	Olson LE, et al.^21^https://pubmed.ncbi.nlm.nih.gov/15499018/	https://www.jax.org/strain/005383
Dp(16Lipi-Zbtb21)1Yey https://www.informatics.jax.org/marker/MGI:3714521	Dp(16)1Yey	Duplication	Cre-mediated chromosomal rearrangement on Mmu16 between loxP sites in regions proximal to D930038D03Rik (*Lipi*) and distal to *Zfp295* (*Zbtb21*) resulted in the duplication of the genes.	B6.129S7	Chr16:75155590-97794739	117	Li Z, et al., 2007 https://pubmed.ncbi.nlm.nih.gov/17412756/	https://www.jax.org/strain/013530
Dp(16Tiam1-Kcnj6)6Yey https://www.informatics.jax.org/marker/MGI:5305761	Dp(16)2Yey	Duplication	Cre-mediated chromosomal rearrangement on Mmu16 between loxP sites in regions proximal to *Tiam1* and distal to *Kcnj6* resulted in the duplication of the genes, including *Tiam1* and *Kcnj6*, in the region that is syntenic with the part of Hsa21q22.	B6;129S7	Chr16:89583999-94798555	52	Liu C, et al.^22^https://pubmed.ncbi.nlm.nih.gov/21442329/	https://www.jax.org/strain/018852
Dp(10Prmt2-Pdxk)2Yey https://www.informatics.jax.org/marker/MGI:4461397	Dp(10)1Yey	Duplication	Cre-mediated chromosomal rearrangement on Mmu10 between loxP sites in regions proximal to *Prmt2* and distal to *Pdxk* results in duplication of the genes, including *Prmt2* and *Pdxk*, in the region that is syntenic with the distal part of Hsa21q22.	B6;129S7	Chr10:76043060-78300782	37	Yu T, et al.^23^https://pubmed.ncbi.nlm.nih.gov/20442137/	https://www.jax.org/strain/013529
Dp(17Abcg1-Rrp1b)3Yey https://www.informatics.jax.org/marker/MGI:4461398	Dp(17)1Yey	Duplication	Cre-mediated chromosomal rearrangement on Mmu17 between loxP sites in regions proximal to *Abcg1* and distal to *Rrp1b* results in duplication of the genes, including *Abcg1* and *Rrp1b*, in the region that is syntenic with the proximal part of Hsa21q22.	B6;129S7	Chr17:31276672-32281839	18	Yu T, et al.^23^https://pubmed.ncbi.nlm.nih.gov/20442137/	https://www.jax.org/strain/013531
Dp(16Tiam1-Il10rb)8Yey https://www.informatics.jax.org/marker/MGI:5562362	Dp(16)3Yey	Duplication	Cre-mediated chromosomal rearrangement on Mmu16 between loxP sites in regions proximal to *Tiam1* and distal to *Il10rb* resulted in the duplication of the genes, including *Tiam1* and *Il10rb*, in the region that is syntenic with the part of Hsa21q22.	129Sv	Not available	17	Liu C, et al.^22^https://pubmed.ncbi.nlm.nih.gov/21442329/	https://www.jax.org/strain/024906
Dp(16Ifnar1-Kcnj6)10Yey https://www.informatics.jax.org/marker/MGI:5562361	Dp(16)4Yey	Duplication	Cre-mediated chromosomal rearrangement on Mmu16 between loxP sites in regions proximal to *Ifnar1* and distal to *Kcnj6* resulted in the duplication of the genes, including *Ifnar1* and *Kcnj6*, in the region that is syntenic with the part of Hsa21q22.	129Sv	Not available	35	Liu C, et al.^22^https://pubmed.ncbi.nlm.nih.gov/21442329/	https://www.jax.org/strain/024908
Dp(17Abcg1-Cbs)1Yah Dp(17Abcg1-Cbs)1Yah	Ts1Yah	Duplication	Cre-mediated chromosomal rearrangement on Mmu17 between loxP inserted in *Abcg1* and downstream of *Cbs* (before *U2af1*) region resulted in the deletion of *Abcg1* and the duplication of the segment homologous and syntenic to the Hsa21q22	B6;129P2	Chr17:31276672-31856173	13	Lopes Pereira P, et al., 2009 https://pubmed.ncbi.nlm.nih.gov/19783846/	https://www.infrafrontier.eu/emma/strain-search/straindetails/?q=1987
Dp(16Hspa13-App)2Yah Dp(16Hspa13-App)2Yah	Ts2Yah/Dp2Yah	Duplication	Cre-mediated chromosomal rearrangement on Mmu16 between loxP inserted in *Hspa13* and *App* resulted in the inactivation of both flanking genes and the duplication of the segment homologous to Hsa21q11-q21	B6;129P2	Chr16:75552078-84970840	19	Brault V, et al., 2015 https://pubmed.ncbi.nlm.nih.gov/25803843/	https://www.infrafrontier.eu/emma/strain-search/straindetails/?q=2101
Dp(10Col6a1-Cstb)3Yah	Ts3Yah/Dp3Yah	Duplication	Cre-mediated chromosomal rearrangement on Hsa16 between loxP inserted in *Cstb* and *Col6a1* resulted in the inactivation of both flanking genes and the duplication of the segment homologous to Hsa21	B6	–	25	*Unpublished*	Herault’s lab
Dp(10Prmt2-Cstb)4Yah	Ts4Yah/Dp4Yah	Duplication	Cre-mediated chromosomal rearrangement on Mmu16 between loxP inserted in *Cstb* and *Prmt2* resulted in the inactivation of both flanking genes and the duplication of the segment homologous to Hsa21	B6	–	36	*Unpublished*	Herault’s lab
Dp(16App-Runx1)5Yah	Ts5Yah/Dp5Yah	Duplication	Cre-mediated chromosomal rearrangement on Mmu16 between loxP inserted in *App* and *Runx1* resulted in the inactivation of both flanking genes and the duplication of the segment homologous to chromosome 21	B6	Chr16:84954440-92826149	60	Duchon A et al., 2021 https://pubmed.ncbi.nlm.nih.gov/33693642/	Herault’s lab
Dp(16*Samsn1-Cldn17*)7Yah	Dp(16)7Yah	Duplication	Cre-mediated chromosomal rearrangement on Mmu16 between *Samsn1* and *Cldn17*	B6	Chr16:75655681-88303866	38	Ahumada Saavedra JT et al., 2024 https://pubmed.ncbi.nlm.nih.gov/40982554/	Herault’s lab
Dp(16*Tiam1-Clic6*))8Yah	Dp(16)8Yah	Duplication	Cre-mediated chromosomal rearrangement on Mmu16 between *Tiam1* and *Clic6*.	B6	Chr16:89583999-92338129	44	Ahumada Saavedra JT et al., 2024 https://pubmed.ncbi.nlm.nih.gov/40982554/	Herault’s lab
Dp(16*Cldn17-Brwd1*))9Yah	Dp(16)9Yah	Duplication	Cre-mediated chromosomal rearrangement on Mmu16 between *Cldn17* and *Brwd1*	B6	Chr16:88302695-95883726	73	Ahumada Saavedra JT et al., 2024 https://pubmed.ncbi.nlm.nih.gov/40982554/	Herault’s lab
Dp(16*Tmprss15-Setd4*)10Yah	Dp(16)10Yah	Duplication	Cre-mediated chromosomal rearrangement on Mmu16 between *Tmprss15* and *Setd4*	B6	Chr16:78749896-93400951	75	Ahumada Saavedra JT et al., 2024 https://pubmed.ncbi.nlm.nih.gov/40982554/	Herault’s lab
Dp(16*Tmprss15-Grik1*)11Yah	Dp(16)11Yah	Duplication	Cre-mediated chromosomal rearrangement on Mmu16 between *Tmprss15* and *Grik1*	B6	Chr16:78749896-88087153	25	Ahumada Saavedra JT et al., 2024 https://pubmed.ncbi.nlm.nih.gov/40982554/	Herault’s lab
Dp(16*Tmprss15-Zbtb21*)12Yah	Dp(16)12Yah	Duplication	Cre-mediated chromosomal rearrangement on Mmu16 between *Tmprss15* to *Zfp295*	B6	Chr16:78749896-97794739	121	Ahumada Saavedra JT et al., 2024 https://pubmed.ncbi.nlm.nih.gov/40982554/	Herault’s lab
Dp(16*Cldn17-Vps26c*(*Dyrk1a*KO))13Yah	Dp(16)13Yah	Duplication	Cre-mediated chromosomal rearrangement on Mmu16 between *Cldn17* to *Vps26c*, up to the sequence of *Dyrk1a* which is inactivated	B6	Chr16:88302695-94327488	63	Ahumada Saavedra JT et al., 2024 https://pubmed.ncbi.nlm.nih.gov/40982554/	Herault’s lab
Dp(16Lipi-Zbtb21)1TybEmcf https://www.informatics.jax.org/allele/MGI:5703798	Dp1Tyb	Duplication	Cre-mediated chromosomal rearrangement on Mmu16 to duplicate the region between two loxP sites inserted proximal to *Lipi* and distal to *Zbtb21*.	B6.129P2	Chr16:74930370-97982380	117	Lana-Elola, E. et al.^24^https://pubmed.ncbi.nlm.nih.gov/26765563/	https://www.infrafrontier.eu/emma/strain-search/straindetails/?q=10565 https://www.jax.org/strain/037183
Dp(16Mis18a-Runx1)2TybEmcf Dp(16Mis18a-Runx1)2TybEmcf	Dp2Tyb	Duplication	Cre-mediated chromosomal rearrangement on Mmu16 to duplicate the region between two loxP sites inserted proximal to *Mis18a* and distal to *Runx1*.	B6.129P2	Chr16:90563769-93062456	32	Lana-Elola, E. et al.^24^https://pubmed.ncbi.nlm.nih.gov/26765563/	https://www.infrafrontier.eu/emma/strain-search/straindetails/?q=10558
Dp(16Mir802-Zbtb21)3TybEmcf Dp(16Mir802-Zbtb21)3TybEmcf	Dp3Tyb	Duplication	Cre-mediated chromosomal rearrangement on Mmu16 to duplicate the region between two loxP sites inserted proximal to *Mir802* and distal to *Zbtb21*.	B6.129P2	Chr16:93054020-97982380	37	LLana-Elola, E. et al.^24^https://pubmed.ncbi.nlm.nih.gov/26765563/	https://www.infrafrontier.eu/emma/strain-search/straindetails/?q=10559
Dp(16Mir802-Dscr3)4TybEmcf Dp(16Mir802-Vps26c)4TybEmcf	Dp4Tyb	Duplication	Cre-mediated chromosomal rearrangement on Mmu16 to duplicate the region between two loxP sites inserted proximal to *Mir802* and distal to *Dscr3*.	B6.129P2	Chr16:93054020-94546849	14	Lana-Elola, E. et al.^24^https://pubmed.ncbi.nlm.nih.gov/26765563/	https://www.infrafrontier.eu/emma/strain-search/straindetails/?q=10560
Dp(16Dyrk1a-B3galt5)5TybEmcf Dp(16Dyrk1a-B3galt5)5TybEmcf	Dp5Tyb	Duplication	Cre-mediated chromosomal rearrangement on Mmu16 to duplicate the region between two loxP sites inserted proximal to *Dyrk1a* and distal to *B3galt5*.	B6.129P2	Chr16:94538615-96331804	12	Lana-Elola, E. et al.^24^https://pubmed.ncbi.nlm.nih.gov/26765563/	https://www.infrafrontier.eu/emma/strain-search/straindetails/?q=10561
Dp(16Igsf5-Zbtb21)6TybEmcf Dp(16Igsf5-Zbtb21)6TybEmcf	Dp6Tyb	Duplication	Cre-mediated chromosomal rearrangement on Mmu16 to duplicate the region between two loxP sites inserted proximal to *Igsf5* and distal to *Zbtb21*.	B6.129P2	Chr16:96327324-97982380	11	Lana-Elola, E. et al.^24^https://pubmed.ncbi.nlm.nih.gov/26765563/	https://www.infrafrontier.eu/emma/strain-search/straindetails/?q=10562
Dp(16Mis18a-Il10rb)7TybEmcf	Dp7Tyb	Duplication	Cre-mediated chromosomal rearrangement on Mmu16 to duplicate the region between two loxP sites inserted proximal to *Mis18a* and distal to *Il10rb*.	B6.129P2	Chr16:90563769-91443378	13	Unpublished	Tybulewicz Lab
Dp(16Ifnar1-Runx1)8TybEmcf	Dp8Tyb	Duplication	Cre-mediated chromosomal rearrangement on Mmu16 to duplicate the region between two loxP sites inserted proximal to *Ifnar1* and distal to *Runx1*.	B6.129P2	Chr16:91436064-93062456	19	Unpublished	Tybulewicz Lab
Dp(16Lipi-Hunk)9TybEmcf Dp(16Lipi-Hunk)9TybEmcf	Dp9Tyb	Duplication	Cre-mediated chromosomal rearrangement on Mmu16 to duplicate the region between two loxP sites inserted proximal to *Lipi* and distal to *Hunk*.	B6.129P2	Chr16:74930370-90577148	48	Lana-Elola, E. et al.^24^https://pubmed.ncbi.nlm.nih.gov/26765563/	https://www.infrafrontier.eu/emma/strain-search/straindetails/?q=10565
**Deletion Models**								
Del(16Cbr1-Fam3b)1Rhr Del(16Cbr1-Fam3b)1Rhr	Ms1Rhr	Deletion	Cre mediated chromosomal rearrangement on Mmu16 between loxP sites in regions proximal to *Cbr1* and distal to *Fam3b* results in deletion of the genes, including *Cbr1* and *Fam3b*, in the region that is syntenic with the distal part of Hsa21q22	B6C3	Chr16:93404725-97306136	29	Olson LE, et al.^21^https://pubmed.ncbi.nlm.nih.gov/15499018/	https://www.jax.org/strain/005654
Del(10Prmt2-Pdxk)4Yey Del(10Prmt2-Pdxk)4Yey	Df(10)1Yey	Deletion	Cre mediated chromosomal rearrangement on Mmu10 between loxP sites in regions proximal to *Prmt2* and distal to *Pdxk* resulted in deletion of the genes, including *Prmt2* and *Pdxk*, in the region that is syntenic with the distal part of Hsa21q22.	Mixed 129S7 and 129S1 background.	Chr10:76043060-78300782	37	Yu T, et al., 2010 https://pubmed.ncbi.nlm.nih.gov/20512340/	https://www.jax.org/strain/017439
Del(17Abcg1-Rrp1b)5Yey Del(17Abcg1-Rrp1b)5Yey	Df(17)1Yey	Deletion	Cre mediated chromosomal rearrangement on Mmu17 between loxP sites in regions proximal to *Abcg1* and distal to *Rrp1b* resulted in the deletion of the genes, including *Abcg1* and *Rrp1b*, in the region that is syntenic with the proximal part of Hsa21q22.	Mixed 129S7 and 129S1 background.	Chr17:31276672-32281839	18	Yu T, et al., 2010 https://pubmed.ncbi.nlm.nih.gov/20512340/	https://www.jax.org/strain/017440
Del(16Tiam1-Kcnj6)7Yey Del(16Tiam1-Kcnj6)7Yey	Df(16)2Yey	Deletion	Cre mediated chromosomal rearrangement on Mmu16 between loxP sites in regions proximal to *Tiam1* and distal to *Kcnj6* resulted in the deletion of the genes, including *Tiam1* and *Kcnj6*, in the region that is syntenic with the part of Hsa21q22.	Mixed 129S7 and 129S1 background.	Chr16:89583999-94798555	52	Liu C, et al.^22^https://pubmed.ncbi.nlm.nih.gov/21442329/	https://www.jax.org/strain/018853
Del(16Tiam1-Il10rb)9Yey Del(16Tiam1-Il10rb)9Yey	Df(16)3Yey	Deletion	Cre mediated chromosomal rearrangement on Mmu16 between loxP sites in regions proximal to *Tiam1* and distal to *Il10rb* resulted in the deletion of the genes, including *Tiam1* and *Il10rb*, in the region that is syntenic with the part of Hsa21q22.	Mixed 129S7 and 129S1 background.	Not available	17	Liu C, et al.^22^https://pubmed.ncbi.nlm.nih.gov/21442329/	https://www.jax.org/strain/024907
Del(16Ifnar1-Kcnj6)11Yey Del(16Ifnar1-Kcnj6)11Yey	Df(16)4Yey	Deletion	Cre mediated chromosomal rearrangement on Mmu16 between loxP sites in regions proximal to *Ifnar1* and distal to *Kcnj6* resulted in the deletion of the genes, including *Ifnar1* and *Kcnj6*, in the region that is syntenic with the part of Hsa21q22.	Mixed 129S7 and 129S1 background.	Not available	35	Liu C, et al.^22^https://pubmed.ncbi.nlm.nih.gov/21442329/	https://www.jax.org/strain/024908
Del(16Setd4-Kcnj6)12Yey Del(16Setd4-Kcnj6)12Yey	Df(16)5Yey	Deletion	Cre mediated chromosomal rearrangement on Mmu16 between loxP sites in regions proximal to *Setd4* and distal to *Kcnj6* resulted in the deletion of the genes, including *Setd4* and *Kcnj6*, in the region that is syntenic with the part of Hsa21q22.	B6;129S7	Not available	15	Jiang X, et al., 2015 https://pubmed.ncbi.nlm.nih.gov/26374847/	https://www.jax.org/strain/028289 (In combination with Dp(16)1Yey)
Del(16Kcnj15-Mx2)13Yey Del(16Kcnj15-Mx2)13Yey	Df(16)6Yey	Deletion	Cre mediated chromosomal rearrangement on Mmu16 between loxP sites in regions proximal to *Kcnj15* and distal to *Mx2* resulted in the deletion of the genes, including *Kcnj15* and *Mx2*, in the region that is syntenic with the part of Hsa21q22.	B6.129S7	Not available	16	Jiang X, et al., 2015 https://pubmed.ncbi.nlm.nih.gov/26374847/	https://www.jax.org/strain/028292
Del(16Dyrk1a-Kcnj6)14Yey Del(16Dyrk1a-Kcnj6)14Yey	Df(16)7Yey	Deletion	Cre mediated chromosomal rearrangement on Mmu16 between loxP sites in regions proximal to *Dyrk1a* and distal to *Kcnj6* resulted in the deletion of the genes, including *Dyrk1a* and *Kcnj6*, in the region that is syntenic with the part of Hsa21q22.	B6.129S7	Not available	2	Jiang X, et al., 2015 https://pubmed.ncbi.nlm.nih.gov/26374847/	https://www.jax.org/strain/028290
Del(16Lipi-Ncam2)8Yey/J https://www.informatics.jax.org/allele/MGI:7509496	Df(16)8Yey	Deletion	Cre mediated chromosomal rearrangement on Mmu16 between loxP sites in regions proximal to *Lipi* and distal to *Ncam2* resulted in the deletion of the genes, including *Lipi* and *Ncam2*, in the region that is syntenic with the part of Hsa21q22.	129S7	Not available	15	Xing, X, et al.^25^https://pubmed.ncbi.nlm.nih.gov/37014740/	https://www.jax.org/strain/038567
Del(17Scaf8-Pde10a)2Yey/J https://www.informatics.jax.org/allele/MGI:7509492	Df(17)2Yey	Deletion	Cre mediated chromosomal rearrangement on Mmu17 between loxP sites in regions proximal to *Scaf8* and distal to *Pde10a* resulted in the deletion of the genes, including *Scaf8* and *Pde10a*.	B6;129S7	Not available	0	Xing, X, et al.^25^https://pubmed.ncbi.nlm.nih.gov/37014740/	https://www.jax.org/strain/038566
Del(10Prmt2-Col6a1)1Yah Del(10Prmt2-Col6a1)1Yah	Ms1Yah	Deletion	Cre mediated chromosomal rearrangement on Mmu16 between loxP inserted in *Col6a1* and *Prmt2* resulted in the inactivation of both flanking genes and the deletion of the segment homologous to Hsa21q22	B6.129P2	Chr10:76043060-76561878	12	Besson V, et al., 2007https://pubmed.ncbi.nlm.nih.gov/17591625/	https://www.infrafrontier.eu/emma/strain-search/straindetails/?q=1808
Del(17Abcg1-Cbs)2Yah Del(17Abcg1-Cbs)2Yah	Ms2Yah	Deletion	Cre mediated chromosomal rearrangement on Mmu16 between loxP inserted in *Abcg1* and *Cbs* resulted in the inactivation of both flanking genes and the deletion of the segment homologous to Hsa21q22	B6;129P2	Chr17:31276672-31856173	13	Lopes Pereira P, et al. https://pubmed.ncbi.nlm.nih.gov/19783846/	https://www.infrafrontier.eu/emma/strain-search/straindetails/?q=1812
Del(16Hspa13-App)3Yah Del(16Hspa13-App)3Yah	Ms3Yah	Deletion	Cre mediated chromosomal rearrangement on Mmu16 between loxP inserted in *Hspa13* and *App* resulted in the inactivation of both flanking genes and the deletion of the segment homologous to Hsa21q22	B6;129P2	Chr16:75552078-84970840	19	Brault V, et al. https://pubmed.ncbi.nlm.nih.gov/25803843/	https://www.infrafrontier.eu/emma/strain-search/straindetails/?q=2100
Del(10Prmt2-Cstb)4Yah https://www.informatics.jax.org/allele/MGI:4868385	Ms4Yah	Deletion	Cre mediated chromosomal rearrangement on Mmu16 between loxP inserted in *Cstb* and *Prmt2* resulted in the inactivation of both flanking genes and the deletion of the segment homologous to Hsa21q22	B6;129P2	Chr10:76043060-78263456	36	Duchon A, et al. https://pubmed.ncbi.nlm.nih.gov/18757940/	https://www.infrafrontier.eu/search?keyword=EM:02099
Del(16App-Runx1)5Yah Del(16App-Runx1)5Yah	Ms5Yah	Deletion	Cre mediated chromosomal rearrangement on Mmu16 between loxP inserted in *App* and *Runx1* resulted in the inactivation of both flanking genes and the deletion of the segment homologous to Hsa21q22	B6J	Chr16:84749554-92622962	60	Raveau M, et al. https://pubmed.ncbi.nlm.nih.gov/22693452/	Herault’s lab

**b. Rat Models**								

**Duplication Models**								
Dp(11Lipi-Zfp295)1Yah	Dp(Rno11)	Duplication	CRISPR-mediated chromosomal rearrangement	Sprague Dawley	Chr11:26077978-38457373	113	Birling M-C. et al. https://pubmed.ncbi.nlm.nih.gov/28266534/	Herault’s lab
Dp(20Umodl1-Prmt2)2Yah	Dp(Rno20)	Duplication	CRISPR-mediated chromosomal rearrangement	Sprague Dawley	Chr20:11825676-15366940	74	Birling M-C. et al. https://pubmed.ncbi.nlm.nih.gov/28266534/	Herault’s lab
Dp(11Dyrk1a)5Yah	Dp(Dyrk1a)	Duplication	CRISPR-mediated chromosomal rearrangement	Sprague Dawley	Chr11:34842454-34963976	Dyrk1a	Birling M-C. et al. https://pubmed.ncbi.nlm.nih.gov/28266534/	Herault’s lab
Dp(11Dyrk1a)6Yah	Dp(Dyrk1a)	Duplication	CRISPR-mediated chromosomal rearrangement	Sprague Dawley	Chr11:34791993-35024196	Dyrk1a	Birling M-C. et al. https://pubmed.ncbi.nlm.nih.gov/28266534/	Herault’s lab
Dp(20Cbs)7Yah	Dp(Cbs)	Duplication	CRISPR-mediated chromosomal rearrangement	Sprague Dawley	Chr20:12550129-12574252	Cbs	Birling M-C. et al. https://pubmed.ncbi.nlm.nih.gov/28266534/	Herault’s lab
**Deletion Models**								
Del(11Lipi-Zfp295)3Yah	Del(Rno20)	Deletion	CRISPR-mediated chromosomal rearrangement	Sprague Dawley	Chr20:11825676-15366940	74	Birling M-C. et al. https://pubmed.ncbi.nlm.nih.gov/28266534/	Herault’s lab
Del(11Dyrk1a)4Yah	Del(Dyrk1a)	Deletion	CRISPR-mediated chromosomal rearrangement	Sprague Dawley	Chr11:34842454-34963976	Dyrk1a	Birling M-C. et al. https://pubmed.ncbi.nlm.nih.gov/28266534/	Herault’s lab
**Transchromosomic Models**								
Tc(HSA21)1Kaz	TcHSA21rat	Transchromosomic	First transchromosomic rat model of Down syndrome. Contains a freely segregating, EGFP-labeled human chromosome 21 (HSA21) with >93% of HSA21 protein-coding genes. Maintained via female transmission on Wistar background.	Wistar (Crlj:WI)	bGRCh38/RefSeq NC_000021.9 (HSA21): loxP insertion 13021348–13028858; deletions (WGS): Del1 17843345–23995878; Del2 28195594–30325968.	145 (The number of orthologous PCGs is 145 after subtracting 17 PCGs in 21p, 46 KRTAPs, and 6 human-specific PCGs from the total number of 214.)	Kazuki Y, et al.^26^https://pubmed.ncbi.nlm.nih.gov/35077668/	Contact authors (Tottori University/Johns Hopkins)

All coordinates based on Mouse GRCm39 and Human GRCh38 unless otherwise noted. This excludes the non-transchromosomic Rat models.

Link to a Live and updatable web database based on this table: Database.

Link to associated GitHub page: GitHub repository.

a Note: Tc1 count includes duplications and anticipated single-copy genes.

b Note: TcMAC21 and TcHSA21rat HSA21 coordinates are reported on Human GRCh38/RefSeq NC_000021.9; WGS mapping in Kazuki et al. used GRCh38.p13 Primary Assembly.

Given the multiplicity of preclinical models of DS, making informed decisions regarding the appropriateness of a model towards answering a specific research question is an important exercise. This primer aims to serve as a practical guide for researchers entering or expanding their work in preclinical DS research using rodent models, particularly mice and rats. We provide an overview of the currently available models, highlighting their genetic composition and phenotypic features. We also discuss strategies for leveraging DS mouse models towards enhancing the robustness and effectiveness of both forward and reverse translational research.

## Main text of the primer

### The genetics of Down syndrome, current rodent models, and their challenges

The extra genetic material present in individuals with DS disrupts the normal balance of gene products, leading to widespread transcriptional perturbations and a range of phenotypic outcomes, including intellectual disability, locomotor defects, and other systemic features that appear throughout life such as cardiac and autoimmune deficits, and early-onset Alzheimer’s disease (AD). The power of rodent genetics as a methodology has enabled us to break down Hsa21 into triplicated candidate regions of interest, and to identify individual dosage-sensitive genes, to assess their contribution to specific phenotypes.

All DS models have been based on regions either from Hsa21 itself (transchromosomic mice) or with homology to Hsa21 in the rodent genome; the first models created and still the most prevalent are those using Hsa21-orthologous regions of the mouse genome. However, a major challenge in modeling DS in rodents is the lack of complete synteny (conserved gene order) between human Hsa21 and any single mouse or rat chromosome. Hsa21 is syntenic with regions on Mmu 16, 10, and 17, containing ∼105, 37, and 19 protein-coding genes^20^ respectively, not including unrelated keratin-associated genes.^29^ As a result, the genes on Hsa21 are distributed across these three mouse chromosomes, complicating the development of models that accurately recapitulate the full trisomy observed in humans.

The first widely used mouse model was the Ts65Dn strain,^30^ which carries an additional chromosome and is trisomic for an Hsa21-orthologous segment of Mmu16 containing 132 protein-coding genes. Although there had been previous attempts at using mouse models to understand DS mechanisms, this model opened the door for the use of mouse genetics to understand genotype-phenotype relationships in DS, helping to identify driver genes and test therapeutic concepts. However, Ts65Dn mice additionally have a third copy of a region of Mmu17 that has no synteny to Hsa21, but contains 46 protein coding genes.^31^^,^^32^ The extra copy of these 46 genes could contribute to phenotypes in Ts65Dn mice but have no genetic relevance to trisomy 21. A new model, Ts66Yah, has been derived from Ts65Dn mice to remove the extra copy of the irrelevant non-Hsa21-orthologous region of Mmu17, leaving a third copy of only the Hsa21-orthologous region of Mmu16.^18^ This creates a model with improved genetic validity that still has an extra chromosome.

Other models that had a seminal influence on the field were created using the then novel technique of chromosome engineering. These three new mouse strains carry the entire Hsa21 regions of orthology from Mmu16, 17, or 10, in three copies, Dp(16)1Yey, Dp(17)1Yey, Dp(10)1Yey mice.^23^ These mouse strains have a tandem duplication of the orthologous regions and no extra chromosome. More recently, Dp1Tyb mice were reported which have been engineered to have three copies of the same region of Mmu16 as Dp(16)1Yey mice.^25^ These mouse strains have been the basis of many studies, for example, in dissecting learning and memory deficits, congenital heart defects and craniofacial defects and confirming the importance of gene dosage in the pathophysiology of DS.^25^^,^^33^ A powerful ‘zoo’ of chromosome-engineered mouse strains carrying smaller regions of homology to Hsa21, across Mmu16, Mmu17 and Mmu10, now exists to help pinpoint key dosage sensitive regions and genes involved in DS phenotypes.^34^ For example, models, such as Ts1Rhr, Dp1Tyb - Dp12Tyb, Dp(16)1Yey - Dp14Yey, Dp1Yah - Dp13Yah, carry duplications of smaller, specific regions of Hsa21 synteny.^34^ These mouse strains have been crucial for dissecting the contribution of individual genes or small gene clusters to DS phenotypes, revealing the complexity of genetic interactions and the combinatorial nature of DS features.

An alternative approach to modelling DS came from creating transchromosomic lines that carry a freely segregating copy of Hsa21, as discussed below. Such mice provide a more complete representation of human trisomy because they carry the DNA sequence of Hsa21 including non-coding elements, but are technically challenging to create and the current models do not carry a complete Hsa21.^19^^,^^20^ They have the advantage that Hsa21 sequences are present in the mouse model, but, of course, as with all mouse models these sequences are expressed within a mouse context.

The use of all these models has shown that DS is a multigenic disorder, with different genes contributing to different aspects of the phenotype. The variability in phenotypic expression is influenced by both the specific genes that are triplicated and the genetic background of the models. Future research is focused on refining models to better mimic human DS, exploring gene-gene and gene-environment interactions, and developing targeted therapies based on the identification of dosage-sensitive genes and pathways.

Research with model organisms underscores the importance of mouse models in elucidating the genetic basis of DS, the critical role of gene dosage, the challenges posed by homology over three chromosomes, and the ongoing efforts to develop more accurate and informative models for research and therapeutic development.

### Transchromosomic rodent models of DS

DS exhibits significant phenotypic variability, including in a range of intellectual disabilities, in the age of onset for early-onset AD, and incomplete penetrance and variable severity of congenital malformations of heart and gastrointestinal tract, hematological and endocrine anomalies, skeletal abnormalities, and immune dysregulation. Since Hsa21 carries more than 500 genes (including known and novel protein-coding genes, miRNAs, and other types of RNA),^14^ complex genetic dysfunctions arise from the triplication of dosage-sensitive genes on Hsa21. This results in the appearance of significant pathological changes all over the body. Therefore, animal models of DS must accommodate both developmental disturbances and age-related pathophysiological conditions.

The most frequently used animal to model DS is the mouse, and the chromosome-engineered segmental trisomy models, discussed above, offer the benefit of examining genotype-phenotype relationships by combining individual candidate regions.^23^^,^^34^ However, mouse models cannot contain the full panoply of human non-coding regions or even genes, and therefore the transchromosomic models that carry human DNA, in this respect, may be more accurate genetic models of DS.

The transchromosomic TcMAC21 mouse model has high construct validity.^20^ TcMAC21 mice are not mosaic (the human transchromosome is present in every cell) and contain 93% of Hsa21q protein coding genes that are expressed and regulatable. TcMAC21 mice recapitulate many DS phenotypes including anomalies in heart, craniofacial skeleton and brain, increased APP protein level, hematological abnormalities, increased chromosomal radiosensitivity and impairments in learning, memory and synaptic plasticity.^20^ Further pathological phenotypes have been identified: impaired neurogenesis of prenatal and postnatal neocortex,^35^^,^^36^ an imbalanced neocortical excitation-inhibition ratio,^37^ cerebellar circuit dysfunction and disrupted Purkinje cell organization,^38^ modulation of SHH signaling,^39^ abnormal vascular physiology,^40^ sleep fragmentation,^41^ and hypermetabolism.^42^ All but one (hypermetabolism) of these high-human-concordance phenotypes have been demonstrated in other mouse models of DS. TcMAC21 exhibits several features of hypermetabolism, whereas individuals with DS suffer from metabolic dysregulation suggesting high risk of developing obesity and diabetes.^43^ The reason for this discrepancy between TcMAC21 and DS phenotypes remains an interesting issue to be addressed in the future.

Rats have advantages over mice as disease model animals, especially in neuroscience: larger organ size, easier handling, social behavior, cognitive behavior, addiction, and impulsivity.^44^ Therefore, a DS rat model is expected to yield a different outcome compared to DS mouse models. Individuals with DS have intellectual disability, early-onset AD, and psychiatric comorbidities such as increased rates of autism spectrum disorder and ADHD. Given the advantages of rat models, particularly for neuroscience and behavioral investigations, the transchromosomic TcHSA21rat was developed by means of chromosome transfer of whole HSA21 into rat cells, which could hold human chromosomes stably.^27^ The TcHSA21rat contains >93% of Hsa21 protein coding genes that are expressed and regulatable, similarly to the TcMAC21 mouse. The TcHSA21rat exhibits learning and memory deficits, anxiety, and hyperactivity. TcHSA21rat brain pathology shows a smaller brain size than controls, cerebellar hypoplasia and reduced cerebellar foliation consistent with human DS brain morphology. The TcHSA21rat also exhibits anomalies in craniofacial morphology, heart development, and stature. Anxiety phenotypes and reduced cerebellar foliation are observed prominently for the first time among DS animal models, suggesting the importance of this DS rat model compared to existing DS mouse models.

Transchromosomic DS mouse and rat models were successfully created and exhibited various DS pathological phenotypes. However, for technical reasons arising from chromosome transfer techniques, the transferred Hsa21s in the animals have small deletions. To transfer intact whole Hsa21, the method of chromosome transfer is being improved: human iPSCs are now utilized as a chromosome donor and chromosomes are directly transferred into recipient cells by means of virus envelope protein.^45^ In the future, it can be expected that mouse and rat models of DS carrying intact whole Hsa21 will be available to further refine our investigations of human DS. However, one note of caution is that microcell-mediated chromosome transfer has been shown to induce chromothripsis, i.e., chromosome shattering, which may account for the chromosomal deletions and rearrangements found in transchromosomic mice.^46^ New methods of chromosome manipulation may need to be developed to avoid this potential limitation.

### Effects of an extra chromosome beyond gene dosage in Down syndrome models

Although DS is most often conceptualized as a disorder of altered gene dosage resulting from trisomy 21, the possibility that the presence of an extra chromosome *itself* contributes to DS phenotypes has long been hypothesized. A supernumerary chromosome must be replicated, segregated, and spatially accommodated within the nucleus—processes that could perturb cell-cycle dynamics and genome regulation independently of gene dosage changes. Early experimental support for this concept came from yeast studies in which yeast artificial chromosomes (YACs) carrying mammalian DNA were introduced as additional chromosomes. Because the mammalian genes on these YACs are not expressed in yeast, the resulting cellular defects were attributed to the presence of extra chromosomal material rather than gene dosage effects, providing proof of principle for chromosome-level impacts.^47^

**Figure 2:**
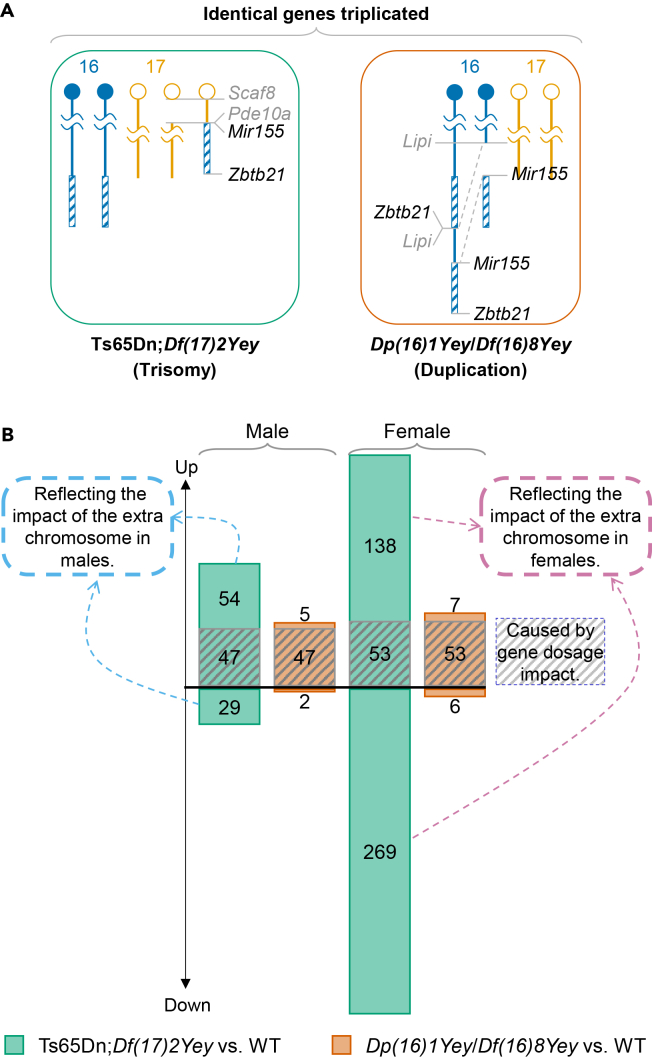
Assessing the contribution of an extra chromosome to DS-related phenotypes (A) Schematic illustration of the Ts65Dn;Df(17)2Yey [i.e., Ts65Dn;Del(17)2Yey] and Dp(16)1Yey/Df(16)8Yey [i.e., Dp(16)1Yey/Del(16)8Yey] compound mouse models. Both models carry identical triplications of Hsa21 gene orthologs within the Mir155 – Zbtb21 interval. Only Ts65Dn;Del(17)2Yey mice retain a freely segregating extra chromosome. 16, Mmu16; 17, Mmu17. (B) RNA-seq analysis of the cerebral cortex revealed that Ts65Dn;Del(17)2Yey mice exhibit altered expression of a substantially larger number of disomic genes compared with Dp(16)1Yey/Del(16)8Yey mice, indicating transcriptomic effects attributable to the presence of a freely segregating extra chromosome beyond those explained by gene dosage alone.

In mammals, this hypothesis has been tested using complementary mouse models of DS^26^: Ts65Dn mice carry an extra, freely segregating chromosome that includes Hsa21-orthologous regions, whereas Dp(16)1Yey mice harbor three copies of the corresponding segment without an increase in chromosome number. To directly assess the contribution of an extra chromosome independent of gene dosage, compound models were generated with the aid of chromosome engineering: Ts65Dn;Df(17)2Yey and Dp(16)1Yey/Df(16)8Yey (Figure 2A). These models normalize the dosage of Hsa21 orthologs while differing in chromosome number, thereby allowing phenotypic and molecular differences to be attributed to the presence of a freely segregating extra chromosome.

Behavioral analyses of these matched compound models revealed both shared and distinct phenotypes. Deficits in nesting behavior were observed in both models, suggesting effects driven by triplication of Hsa21 orthologs. In contrast, impairments in T-maze performance were detected in Ts65Dn;Df(17)2Yey mice but not in Dp(16)1Yey/Df(16)8Yey mice, implicating the extra chromosome in specific cognitive deficits beyond gene dosage.

Transcriptomic analyses of the cerebral cortex further supported a chromosome-level effect. While both models showed elevated expression of a subset of triplicated Hsa21 orthologs, Ts65Dn;Df(17)2Yey mice displayed a markedly greater number of differentially expressed disomic genes than Dp(16)1Yey/Df(16)8Yey mice, as summarized in Figure 2B. A similar pattern was observed in brainstem samples. Together, these findings indicate that the presence of an extra chromosome exerts significant transcriptomic effects beyond those attributable to gene dosage alone.

Trisomy 21 has been associated with disruptions in nuclear lamina organization and changes in three-dimensional genome architecture.^48^^,^^49^ The matched compound models described here provide an appropriate platform to dissect how such chromosome-level effects contribute to DS phenotypes beyond gene dosage.

### Benchmarks for genetic rigor and model selection criteria

The selection and use of rodent models of DS is a genetics-first approach because DS phenotypes are caused by trisomy 21.^50^ This approach has challenges, primarily as noted, because Hsa21-orthologous genes are found in the rat on Rno11, and 20, and in mice on Mmu10, 16, and 17. To maximize construct validity, criteria that should be considered include conservation of genetic material found on Hsa21 in rodent models (numbers of triplicated genes), source of the extra genetic material (rodent or human), and nature of the triplication (freely segregating extra chromosome, duplication of gene segments, or transgenes).^26^^,^^51^^,^^52^ Genetic rigor should also consider the genetic background (inbred, advanced intercross, or more diverse) and allelic differences between different strains of mice (for example, Ts65Dn 001924 or 005252 and Dp(16)1Yey or Dp1Tyb).^28^^,^^53^

Ideally, a rodent model should have as many of the Hsa21 homologs as possible in three copies on a freely segregating chromosome that mimics trisomy 21 in humans. Models in which Hsa21 has been integrated into rodent cells to produce the trisomy have more genes in three copies but may have genomic loss or changes and some human genes may function differently in a rodent.^19^^,^^20^^,^^27^^,^^54^ In rodent models, preventing genetic drift is important and may be done through frequent reintroduction of rodent models from well-established genetic repositories, including the Jackson Laboratory, into an established rodent colony.

DS rodent models should also be selected for face (phenotypic and mechanistic) validity based on developmental and adult stages.^55^ For example, in the Ts65Dn DS mouse model, transmission of the extra chromosome usually happens through the female trisomic parent which generally does not replicate what occurs in most humans with Ts21. The timing of brain development (prenatal and postnatal) differs in humans and rodents and comparisons between model systems should be included. In rodents, the number of offspring in a litter may influence size of offspring and acquired characteristics due to a larger or smaller size. Additionally, the parent of origin that provides the additional genetic material has been shown to influence phenotypic outcomes.^56^ These factors should be considered when selecting a model and acknowledged in experimental descriptions.

Face validity encompasses both mechanistic and phenotypic criteria for rodent models of DS. Some but not all molecular pathways are conserved between humans, rats, and mice. Rodent models are effective tools to elucidate biological mechanisms that produce the pathophysiology of translatable features in humans.^50^ In addition to conserved cellular and tissue mechanisms leading to disease, phenotypes between rodent models and humans should be comparable, and a model that effectively exhibits a particular phenotype should be chosen. Such phenotypic and mechanistic validity has been illustrated by DS craniofacial features in mice and humans and should also be extended to accurate replication of learning and memory phenotypes. The proper criteria to select rodent models of DS should also include predictive validity for the etiology of a particular trait that may lead to a proposed treatment or may include similar biomarkers in humans and rodents indicative of that pathology.^57^^,^^58^ If a phenotype is being characterized for the first time, it is best to use at least two different rodent models and/or replicate findings in two different laboratory settings.^28^

### Neurobiology and behavior in Down syndrome mouse models

Persistent cognitive and motor disability during infancy and childhood and, in adulthood, a high burden of AD neuropathology^1^ make DS one of the most consequential neurodevelopmental conditions across the lifespan. DS rodent models enable causal linkage of gene-dosage imbalance to early circuit assembly and to later-life vulnerabilities that influence ‘cognitive reserve’ and dementia expression.^17^^,^^59^ Because trisomy perturbs hundreds of genes and downstream pathways, no single model can fully represent the complexity of atypical behavior in DS.

**Figure 3:**
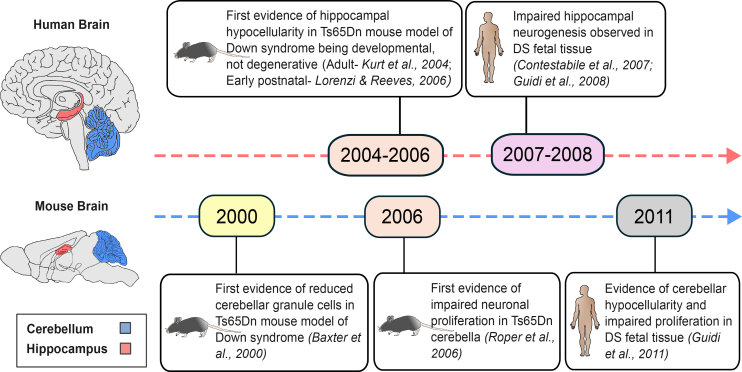
Small Model, Big Insights: Mechanistic Discovery in Down Syndrome Using the Mouse Brain Rodent models have led to important discoveries in Down syndrome. The translational impact of rodent models of DS is especially evident at the neurodevelopmental and cellular-anatomical levels. The key discovery of impaired neurogenesis in DS was first observed in the hippocampus and cerebellum in rodent models and then confirmed in humans. While early clinical literature reports volumetric deficits and cellular changes, including altered spine distribution, in the hippocampus,^63^^,^^64^^,^^65^^,^^66^^,^^67^ hypocellularity in specific sub-regions was shown to be caused by developmental impairment in neuronal proliferation observed in the Ts65Dn mouse model of Down syndrome.^68^^,^^69^ This would later accord with data from DS fetal tissue, as reported by Contestabile et al. (2007) and Guidi et al. (2008) – specifically – reduced volume and cell number in the dentate gyrus, hippocampus, and parahippocampal gyrus, fewer neuronal-phenotype cells, fewer Ki-67-positive proliferating cells, and increased apoptotic cell death.^70^^,^^71^ More recent MRI work in individuals with Down syndrome has also shown selective reductions in bilateral hippocampal subfields, including CA1, dentate gyrus, and hippocampal tail, together with altered hippocampal functional connectivity.^72^ The Ts65Dn model has also been instrumental in identifying a drastic reduction in the density of cerebellar granule cells as the underlying cause of cerebellar hypoplasia, which is a key feature of DS.^73^^,^^74^ Subsequent studies found defects in Sonic Hedgehog signaling as the mechanism of impaired proliferation and explored a restorative treatment strategy with the Sonic Hedgehog agonist.^61^^,^^75^ A decade following the Baxter et al. study, a study by Guidi et al. found similar hypocellularity in DS fetal tissue aged 17-21 week due to impaired proliferation of precursors from both cerebellar neurogenic regions.^76^ These studies serve as testimony to the power of rodent models in predicting and guiding clinical findings in DS. Furthermore, it underscores the value of forward and reverse translational strategies in unravelling the pathophysiological underpinnings of DS.

Across DS models, convergent neuroanatomical signatures include reduced total brain volume with disproportionate involvement of cerebellar and hippocampal/cortical systems,^16^ a finding that has high human concordance^60^ (Figure 3). Cerebellar hypoplasia, among the most robust Ts65Dn mouse findings, is mechanistically linked to impaired early postnatal expansion of cerebellar granule cell precursors and reduced granule neuron density.^61^ Hippocampal and neocortical circuitry abnormalities often involve altered synaptic structure and excitation-inhibition balance, consistent with deficits in episodic-like learning and executive function.^62^ Together, these convergent neuroanatomical alterations provide a foundation for interpreting the behavioral phenotypes observed in DS models. Accordingly, careful behavioral assessment is essential to disentangle the contributions of primary cognitive dysfunction from secondary performance limitations arising from motor or sensory deficits.

Robust neurobehavioral batteries using DS mouse models must distinguish cognitive impairment from performance limitations (hypotonia, sensory deficits, altered anxiety/stress response). An approach in which screening is conducted for (i) sensorimotor screening (locomotion, gait, strength; vision/hearing checks where feasible), (ii) motor coordination and balance (rotarod, beam tasks), and (iii) cognition with quantified motor and motivational demands.^52^ Dp(16)1Yey mice, for example, show impaired motor coordination on the rotarod and deficits across hippocampus-dependent tasks including contextual fear conditioning and spatial learning in the Morris water maze, as well as novel object recognition and spontaneous alternation.^33^ Such cross-domain profiles mirror the clinical co-occurrence of motor delay and cognitive impairment in DS and help avoid attributing poor task performance to cognition when the limiting factor is motor output.

Translational relevance increases when DS rodent phenotyping targets domains that affect day-to-day function and may interact with dementia risk factors including sleep/circadian dysregulation,^59^ anxiety,^77^ and social-communication differences.^78^ Aged Dp(16)1Yey mice show increased wake time at the expense of non-REM sleep and altered EEG spectral power distributions, providing an interpretable bridge from network physiology to a highly prevalent clinical phenotype in DS.^79^ These measures are particularly valuable because they are quantifiable, longitudinal, and closer to human electrophysiology than many conventional maze endpoints. Building on these behavioral and electrophysiological findings, insights from cellular and molecular analyses have begun to reveal the developmental mechanisms underlying these brain-level alterations.

Trisomic cerebellar granule cell precursors show a cell-autonomous deficit in responsiveness to Sonic hedgehog (Shh) mitogenic signaling, constraining postnatal proliferation and producing persistent cerebellar hypocellularity.^80^ This mechanism is actionable: a single neonatal administration of the Smoothened agonist (SAG) in Ts65Dn normalized adult cerebellar morphology and improved performance on spatial learning tasks,^61^ demonstrating that developmental pathway correction has the potential to generate durable circuit- and behavior-level effects. For DS and AD-DS, this is conceptually important: it supports the idea that early-life circuit ‘set points’ may be modifiable and may influence later resilience to neurodegenerative processes.

Cortical dysfunction, particularly within the prefrontal cortex (PFC) networks supporting working memory and executive control, adds a second mechanistic axis beyond hippocampus and cerebellum.^81^ *Ex vivo* physiology in Ts65Dn PFC has identified glutamatergic synaptic alterations in layer V/VI pyramidal neurons and a reshaped plasticity repertoire, including absence of long-term depression under tested induction conditions despite preserved potentiation.^82^ Such findings motivate behavioral endpoints that interrogate executive control and working memory (rather than relying exclusively on hippocampus-centric tasks) and encourage multi-region circuit mapping when linking molecular targets to cognition.

At the hippocampal circuit level, excessive inhibition and impaired long-term potentiation (LTP) remain among the most replicated DS mouse phenotypes. Kleschevnikov *et al.* showed that dentate gyrus LTP is strongly suppressed by increased inhibition in Ts65Dn mice, with reduced effective NMDA receptor activation during induction, providing a mechanistic substrate for hippocampus-dependent learning and memory deficits.^83^ In parallel, adult plasticity alterations extend to neurogenesis and cell-fate programs: in Ts1Cje mice, systematic analyses of adult neurogenesis revealed reduced neural progenitors and neuroblasts, reduced neuronal output, and increased astrocytic production. These observations support an integrated view in which inhibitory tone, synaptic plasticity rules, and neurogenic capacity jointly constrain learning, and in which sleep/oscillatory biomarkers can serve as intermediate phenotypes linking microcircuit dysfunction to behavior.

From the clinical literature, it is evident that DS is both a neurodevelopmental and a neurodegenerative condition.^84^ The recent application of normative modeling to understand trajectories of brain development and aging provides a robust framework for mapping pathological mechanisms within and across specific populations.^85^ Despite species differences, rodent models are highly amenable to research questions across the lifespan, due to conserved patterns of nervous system development and aging-related changes at the cellular and circuit levels.^16^^,^^17^^,^^86^^,^^87^ Rodent models of DS have been useful in dissecting early impairments caused by disrupted neurogenesis, synaptogenesis, and circuit organization.^16^ Key developmental events that occur prenatally in humans often take place postnatally in rodents, allowing for more accessible investigation - for example, in studies of cerebellar development and associated deficits.^86^ In the TcMAC21 model, defects in neurogenesis and differentiation, along with a reduction in progenitor cells within the neocortex, result in lasting effects on neuronal output and neocortical activity.^35^^,^^36^ Similarly, hippocampal developmental alterations in Ts65Dn mice include a delayed excitatory-to-inhibitory GABA switch, potentially due to reduced neonatal activity and altered chloride homeostasis.^88^ In Dp(16)1Yey mice, evidence indicates that prenatal forebrain development is not substantially altered; however, postnatal delays in growth and developmental milestones have been observed, along with altered cortical cell populations at P15, highlighting the importance of the developmental stage in phenotypic expression.^89^ In contrast, septohippocampal cholinergic neurons do not show early developmental loss but instead exhibit age-related degeneration beginning at 6 months and progressively worsening at 20 months.^90^ In addition, the Tc1 and Dp(16)1Yey mouse models show progressive motor neuron degeneration, with corresponding motor neuron loss also identified in human DS tissue, further supporting the neurodegenerative component of the disorder.^91^

Because distinct tissue-level alterations across the lifespan can manifest similarly in terms of behavioral deficits - for example, memory-associated cognitive deficits - age-specific timepoints and longitudinal approaches are important considerations for experimental design. Such strategies are necessary to link evolving circuit changes to behavioral outcomes over time as well as to disentangle *developmental* from *degenerative* contributions to these outcomes.. Previous reviews have discussed the timing of pathophysiology and therapy in rodent models of DS in much greater detail.^16^^,^^92^

### Benchmarking standards for neurobehavioral phenotyping

Given strong construct-, age-, sex-, and assay-dependence, benchmarking should emphasize transparency and reproducibility. Minimum standards include: explicit trisomic gene content (and any non-syntenic regions), background strain and breeding scheme (including maternal genotype effects), a priori inclusion of both sexes and defined age windows, and reporting of key covariates (body weight, sensory function, locomotor capacity) that influence task performance. Harmonized batteries should span motor coordination, hippocampal learning/memory, PFC/executive function, and sleep/circadian behavior, paired where feasible with circuit readouts (slice plasticity, EEG, fiber photometry). In the context of motor function, it is especially helpful to use automated neurobehavioral tools that distinguish general motor impairment from motor learning so that non-brain-related phenotypes such as muscle strength are taken into account while narrowing down evaluation of behavior associated with specific brain regions. Adopting rigorous reporting frameworks such as ARRIVE 2.0 (randomization, blinding, sample-size rationale, and complete methods reporting) will strengthen cross-laboratory comparability, enable meta-analytic synthesis, and improve the translational value of DS neurobehavioral datasets for DS-AD. Readers are also directed to specific considerations when working with DS rodent models based on ARRIVE guidelines that have been published previously^52^ (under the section critical parameters; subsection: information to include in manuscripts and presentations with DS mouse models).

### Recent insights into craniofacial, skeletal, and cardiac deficits from DS mouse models

Beyond their utility in modeling neurodevelopmental and AD-DS trajectories, DS mouse models offer substantial translational value in dissecting other prominent phenotypes reported in human clinical studies including skeletal and cardiac conditions.^93^

All individuals with DS have skeletal alterations and ∼50% have cardiac deficits. The altered craniofacial appearance is a hallmark of individuals with DS along with short stature and low bone mineral density. Insights into the genetic, cellular, and molecular changes that affect skeletal and cardiac features associated with DS have been made possible using mouse models of DS. Craniofacial alterations, a hallmark feature of individuals with trisomy 21, create a shared appearance between individuals with the condition but also affect how these individuals breathe, eat, and sleep, among other characteristics. Mouse models were shown to exhibit craniofacial alterations that were similar to individuals with DS^94^^,^^95^ and this distinct craniofacial appearance was used to disprove the Down Syndrome Critical Region hypothesis, an assertion that only a region of Hsa21 was important in the majority of DS-associated traits.^21^ The Ts65Dn mouse model defined the prenatal cellular origin of the altered craniofacial structure and implicated the trisomic gene *Dyrk1a* in the formation of craniofacial deficits by affecting neural crest cell (NCC) proliferation, differentiation, and migration.^96^^,^^97^ Analyses using Dp(16)1Yey DS mouse models and a DS mouse mapping panel separating triplicated homologous gene regions on Hsa21 showed that triplicated *Dyrk1a* and *Ripply3* affected NCC proliferation.^98^^,^^99^ Ripply3 likely downregulates non-trisomic *Tbx1* to cause abnormal craniofacial structures.

Skeletal alterations affecting short stature and long bones have been further defined using mouse models of DS. These animals at multiple ages and bone developmental stages have been shown to recapitulate long bone and spine deficits seen in individuals with DS including osteopenic and osteoporotic phenotypes.^100^^,^^101^^,^^102^ Alterations in fracture healing have also been demonstrated in DS mouse models.^103^^,^^104^ Bone in DS mouse models exhibit lower bone volume, bone mineral density, cross-sectional area and other bone integrity and bone microarchitectural parameters. Deficits in osteoblasts (bone-forming), and osteoclasts (bone-resorbing) cells affecting bone composition, often difficult to ascertain from studies in humans with DS because of low numbers,^105^ have been better defined through animal models of DS.^100^^,^^101^ Humans with DS also show a sexual dimorphism in when appendicular skeletal deficits appear; different from typical human skeletal maturation, males with DS generally show skeletal deficits before females,^106^ a feature that has been replicated in DS mouse models and the developmental reasons for these differences are now being examined.^107^^,^^108^^,^^109^ Trisomic *Dyrk1a* seems to affect the male sex, mostly in trabecular bone development.^107^^,^^108^

DS mouse models also have been instrumental in understanding cardiac deficits with DS. Defects of the endocardial cushions including atrioventricular canal defects (AVCD), ventricular septal defects (VSD), atrial septal defects (ASD), patent ductus arteriosus, and tetralogy of Fallot are the most common congenital heart defects (CHD) associated with DS and affect ∼50% of individuals with DS.^110^ DS mouse models have some cardiac abnormalities but do not fully replicate the percentage and types of deficits seen in individuals with DS, likely because they do not have sufficient trisomic genetic content. Ts65Dn and Ts1Cje DS mice show some septal defects and these models have been used to identify DS CHD triplicated *Jam2* and non-triplicated modifier genes including *Creld1* and *Hey2*.^111^^,^^112^ Dp(16)1Yey and Dp(16)4Yey mice exhibited cardiac deficits in ∼25% of embryonic day (E)18.5 embryos and implicated a region containing ∼35 triplicated genes on Mmu16.^24^ The Dp1Tyb DS mouse model showed VSD, ASD, and outflow tract deficits in 50% of E14.5 embryos.^25^ These deficits were linked to triplicated *Dyrk1a* and at least one other triplicated gene that impaired cell proliferation and mitochondrial respiration of cardiomyocytes in the developing Dp1Tyb embryos.^113^ Dp1Tyb embryos showed that increased expression of the triplicated *Hmgn1* shifted myocardium development toward a more ventricular myocardial state, and normalization of *Hmgn1* in Dp1Tyb mice increased the survival of these pups to wild-type levels.^114^ Cardiac deficits may also show developmental effects in the Dp(16)1Yey model. For example, Dp(16)1Yey embryos show increased tissue stiffness, decreased muscularization, cell proliferation, and apoptosis in the endocardial cushions, suggesting a delay in the endocardial to mesenchymal transition that could delay or impair atrioventricular septation.^115^^,^^116^ While Adult Dp(16)1Yey mice show a hypotonicity of the left and right ventricles, evidence for Congenital Heart Disease is lacking. Given that DS mouse models show cardiac deficits are affected by trisomic and non-trisomic genes at differing developmental times and structures, it emphasises the need for assessment of DS-related phenotypes across the lifespan.

### Insights into molecular and cellular pathways

At the molecular level, murine DS models consistently reveal dysregulation of pathways controlling cellular metabolism and proteostasis, with a central role for the mammalian target of rapamycin (mTOR) axis. The mTOR is a serine/threonine kinase that integrates signals from growth factors, nutrients, and cellular stress to regulate protein synthesis, autophagy, cytoskeletal organization, and cell survival. Aberrant mTOR activation is detected early in trisomic brains and is associated with impaired autophagy, altered synaptic plasticity, and disrupted neuronal connectivity.^117^^,^^118^^,^^119^^,^^120^ In particular, studies in Ts65Dn and related mouse models consistently reported hyperactivation of the PI3K/Akt/mTOR axis, with increased mTORC1 signaling detectable at early stages, prior to overt neurodegeneration. This aberrant activation was associated with suppression of autophagy, mediated by inhibition of autophagosome formation, Unc-51 Like Autophagy Activating Kinase 1 (ULK1) and Autophagy-related proteins (Atgs), leading to impaired clearance of misfolded proteins and progressive accumulation of Aβ and hyper-phosphorylated tau into the brain. Thus, murine studies further demonstrated that mTOR hyperactivation represents a reversible and pharmacologically targetable driver of brain dysfunction in DS.^121^^,^^122^ Both prenatal and chronic rapamycin treatment, which is a selective mTORC1 inhibitor, normalized mTOR signaling, restored autophagic flux, corrected synaptic plasticity defects, and improved learning and memory performance in trisomic mice.^118^^,^^123^ In addition, intranasal rapamycin administration in Ts65Dn mice restored insulin signaling and proteostasis while reducing oxidative stress, amyloidogenic APP processing, and tau hyperphosphorylation, resulting in measurable cognitive improvement.^118^^,^^124^ Together, these data support mTOR modulation as a tractable therapeutic target identified through murine experimentation. Consistently, these preclinical findings have motivated the advancement of rapamycin into early-stage clinical evaluation in AD and mild cognitive impairment (MCI), where Phase 1/2 trials are assessing safety and tolerability, with emerging data showing good tolerability.^125^

Beyond mTOR dysregulation, murine models identified brain insulin resistance as a core pathogenic mechanism in DS. Trisomic mice (Ts65Dn) exhibit early and persistent impairments in cerebral insulin signaling, glucose utilization, and mitochondrial bioenergetics, independent of peripheral metabolic alterations.^43^^,^^126^^,^^127^ These defects arise during brain maturation, precede neurodegeneration, and are associated with synaptic dysfunction and cognitive impairment, supporting the classification of insulin resistance as an intrinsic brain phenotype in DS. Importantly, age-stratified analyses across defined developmental stages in trisomic mice enabled the identification of an early temporal window for the onset of brain insulin resistance. This temporal information, not accessible in human studies, provided the experimental rationale for focusing subsequent human investigations on neuronal-derived extracellular vesicles (nEVs) isolated from pediatric cohorts. Analyses of nEV cargo in individuals with DS revealed accumulation of canonical markers of insulin resistance, including increased inhibitory phosphorylation of IRS1 and aberrant activation of the PI3K/Akt/mTOR pathway, in the absence of peripheral metabolic dysfunction.^128^ These vesicle-based molecular signatures closely mirror alterations originally identified in trisomic mouse brains, indicating that insulin signaling defects are established early and persist across the lifespan. nEV profiling further identified alterations in pathways regulating glucose metabolism, mitochondrial function, and synaptic plasticity, supporting their use as minimally invasive biomarkers of brain-specific metabolic dysfunction in DS and providing a mechanistic basis for insulin-targeting interventions.^129^ Consistent with these findings, intranasal administration of the KYCCSRK peptide in Ts2Cje mice restored brain insulin responsiveness, reduced oxidative stress and AD-like pathology, and improved synaptic plasticity, demonstrating that brain insulin resistance can be functionally targeted via intranasal delivery in trisomic models.^129^ Translation of these preclinical observations to humans has begun, with pilot clinical studies showing that intranasal insulin administration in adults with DS is safe and feasible and is associated with preliminary cognitive benefits.^130^

The application of omics approaches further enhanced the translational utility of murine DS models. Transcriptomic and proteomic profiling of trisomic mouse brains revealed widespread dysregulation extending beyond Hsa21 gene products, affecting RNA metabolism, protein folding, stress response pathways, and synaptic organization. Transcriptomic approaches, particularly single-cell RNA sequencing (scRNA-seq), have provided critical insights into cell-type–specific alterations associated with DS and its neurodegenerative trajectory. scRNA-seq analyses of DS fetal brain tissue together with parallel analyses in DS mouse models revealed early loss of ribosomal stoichiometry, mitochondrial dysfunction, and induction of cellular senescence across multiple cell types, including fibroblasts and neural progenitor cells, compared with age-matched controls.^131^ These alterations, first defined and experimentally dissected in trisomic mice, overlap with molecular signatures detected in human DS tissue and are functionally linked to dysregulation of mTOR-dependent processes governing protein synthesis, mitochondrial activity, and cellular proliferation.^132^ Additional studies reported impaired neuronal differentiation and an early imbalance between neuronal and glial populations, indicating that developmental abnormalities observed in human DS brains are recapitulated from early stages in murine models.^133^^,^^134^ Consistently, transcriptomic analyses in DS mouse models identified upregulation of mTOR-related signaling pathways in hippocampal and prefrontal regions, linking molecular alterations defined in vivo to memory-related circuits.^77^ The introduction of spatial transcriptomics further strengthened the translational relevance of murine findings by enabling spatially resolved comparison between species.^135^ Spatial omics studies across human and murine models of AD and DS-associated AD identified species-conserved amyloid-associated gene signatures and region-specific molecular alterations.^136^ However, spatially resolved analyses of mTOR pathway dysregulation remain limited in DS and DS/AD, underscoring the need to extend spatial transcriptomic approaches initially validated in mouse models to the human trisomic brain.^137^

Proteomic analyses provided the most direct evidence of cross-species convergence. Importantly, comparative proteomic analyses of frontal cortex samples from individuals with DS and from trisomic mouse models (Ts66Yah) demonstrated a strong conservation of dysregulated molecular pathways, including energy metabolism, mitochondrial function, proteostasis networks (mTOR signaling, chaperone-mediated autophagy, and unfolded protein response), polyubiquitination profile, redox regulation, and synaptic and neurotransmission-related processes, supporting their relevance as translational targets rather than model-specific artifacts.^9^^,^^138^^,^^139^^,^^140^ Notably, several of these proteostatic and redox-related alterations were first identified in trisomic mouse brains and subsequently confirmed in human DS tissue, highlighting the predictive value of murine models. In parallel, proteomic mapping of oxidative modifications in trisomic mice revealed a close association between aberrant mTOR activation and accumulation of HNE (4-Hydroxynonenal)-modified proteins following rapamycin treatment, further linking molecular mechanisms defined in murine systems to pathways disrupted in the human condition.^124^

### Using rodent models to understand potential sex differences in DS traits

Increasingly, differences in trait manifestation and severity between the sexes of individuals with DS are becoming more recognized. In infants with DS, females as compared to males achieve the majority of developmental milestones significantly earlier; male infants with DS achieve some motor skills earlier than females with DS.^141^ Girls with DS are more typically in the above-average group on intelligence and adaptive function compared to boys; eight-year-old girls had significantly higher developmental age than boys; in adolescents, males exhibited behavioral problems more often than females; in adults, women show higher cognitive abilities compared to men, and the frequency of profound intellectual disability was twice as high in men as women.^142^^,^^143^^,^^144^^,^^145^ Girls with DS show higher rates of CHD, especially with septal defects.^146^ An increased risk of obesity occurs in female children and women with DS and may lead to a higher rate of insulin resistance in females with DS. Females as compared to males with DS have a higher risk of hypertension, ischemic heart disease and cerebrovascular disease.^146^^,^^147^ Bone deficits, including those in the developing tibia, develop earlier in boys as compared to girls with DS, even prior to the surge of sex hormones associated with puberty.^148^^,^^149^ In populations without DS, older females are at risk for increased skeletal deficits; in contrast, males with DS exhibit skeletal deficits before females with DS, although both males and females with DS display skeletal deficits much earlier than normal individuals.^106^^,^^150^^,^^151^^,^^152^ While numerous studies have reported a higher prevalence of AD in women compared to men,^153^ it is not clear whether there are differences in the development of Alzheimer disease (AD) between females and males with DS. In some studies, the development of traits associated with AD occurs earlier in females as compared to males with DS, although differential hormone secretion may influence these differences.^154^^,^^155^^,^^156^

DS rodent models have been effectively used to identify potential sexual dimorphisms in DS-associated traits. Sexual dimorphisms have been observed in many traits in DS model mice that may help to understand similar sex differences in humans. In developmental phenotypes, male Ts65Dn mice showed delays in achieving surface righting, negative geotaxis, cliff aversion, and air righting milestones, whereas female Ts65Dn mice were significantly delayed only in cliff aversion.^55^ There are significant differences between the sexes in the expression of DYRK1A in both bone and brain compartments in DS mouse models.^107^^,^^157^^,^^158^ Almost all trabecular and cortical skeletal deficits were more evident in male as compared to female DS model mice, and trisomic as compared to euploid male mice exhibit skeletal deficits much earlier than females.^109^^,^^159^ Triplicated *Dyrk1a* affects bone in male before female DS mice, has an essential role on trabecular bone deficits in male DS mice, and interacts with other genes to cause cortical bone deficits in male and female DS mice.^107^^,^^108^^,^^160^ When the contribution of the extra chromosome to DS related phenotypes was studied in the Ts65Dn;Df(17)2Yey and Dp(16)1Yey/Df(16)8Yey compound mouse models (Figure 2), there was a sexual dimorphism in the outcomes of the nesting and T maze tests.^26^

DS mouse models have indicated potential behavioral, cellular, and molecular differences between the sexes, but sexual dimorphisms have not been identified in many studies. In Ts65Dn mice, environmental enrichment enhanced circadian spontaneous activity in trisomic males, but it was reduced in females, and enrichment improved spatial learning in Ts65Dn female but not male mice.^161^ Testosterone and adrenocorticotropic hormone (ACTH) levels were not different in male Ts65Dn and control mice, but corticosteroid levels were higher in Ts65Dn mice housed in large groups with and without environmental enrichment.^162^ In a predator exposure test, both trisomic and control female mice showed elevated ACTH and corticosteroid levels, and Ts65Dn mice had reduced (as compared to control mice) responses to predator exposure.^163^ In Ts65Dn mice, female mice had fewer of some basal forebrain cholinergic neurons (BFCN) than males at 5-8 months, and these cholinergic neurons were smaller in Ts65Dn female mice.^164^ Some of these differences may be related to hormonal factors as they interact with trisomic conditions. Female as compared to male Ts65Dn mice exhibited increased soluble Aβ_40_ and Aβ_42_ levels in the basal forebrain between 4 and 8 months of age.^165^ In Dp(16)1Yey mice, sex-related differences in AD related phenotypes connected to APP were not found.^166^ When altered neurodegeneration in the Ts66Yah DS mouse model was examined, sex played a small role in some molecular changes, but these changes did not seem to have a large effect on cognitive phenotypes.^167^ It will be important to continue the use of DS mouse and rat models to understand the impact of sex on DS-related behavioral and degenerative phenotypes. Rodent models have been used to understand the mechanisms of sexual dimorphism in some DS-associated traits, and offer an important tool to understand how, because of differences between the sexes in individuals with DS, sex-specific treatment approaches may be warranted.

### Integrating understanding with cell-, tissue-, and organ-based models

Due to the recent surge in our understanding and use of human-induced pluripotent stem cells (hiPSCs), the number of publications using these cells to advance our understanding of DS has increased significantly. hiPSCs have been used in monolayer tissue culture systems and as three-dimensional organoids to model various aspects of DS^4^^,^^168^ (reviewed in Watson and Meharena, 2023, Gough et al., 2020). Using these methods, numerous clinical phenotypes of DS have been modeled, including heart development, hematopoiesis, and brain development and function (e.g., neurogenesis, axon outgrowth, synapse function, DS-AD).^5^^,^^6^^,^^115^^,^^169^^,^^170^^,^^171^^,^^172^^,^^173^^,^^174^^,^^175^^,^^176^^,^^177^

hiPSCs have also been used in combination with mouse models to create human-mouse chimeras that advance our understanding of DS and DS-AD by potentially allowing for neurons to reach a more mature phenotype (reviewed in Watson and Meharena, 2023, Papetti et al., 2025, Wu et al., 2022b).^6^^,^^178^ For example, using this technique, a foundational study identified multiple differences in synaptic structure and function in DS.^179^ This type of work can also provide insight into the potential use of regenerative medicine to treat cognitive impairment and AD characteristic of DS. However, much about the maturation of these cells and their structural and functional integration remains unclear.

Despite the growing opportunities offered by cell, tissue and organoid-based models, work in the field indicates clear limitations.^180^ When compared to animal models, cellular based models have limitations including simplified multi-cellular complexities, difficulty achieving pure and consistent cell-fate, and the tissues grown do not correspond to developmentally accurate spatial and temporal organ patterning.^181^ Standard 2D tissue cultures contain few interacting cell types, with inaccurate mechanical context due to the culture dish materials.^182^ 3D organoid assemblies, despite having advantages over 2D cultures, have challenges maintaining a consistent morphology over repeated differentiations, as well as, limited maturation and simplified structure and function when compared to *in vivo* models.^183^ As such, congenital defects are challenging to ascertain. Furthermore, the size of organoids is limited to diffusion-based length-scales unless more complex microfluidic devices are introduced.^184^ Finally, from a multi-system perspective, it is challenging to create interconnected organ systems without exponentially increasing setup complexity.^185^

In addition to the inherent cell based limitations, these could be further exaggerated in DS because of the natural biological variability within individuals with DS.^186^ Furthermore, behavioral tests cannot be conducted using cell- and tissue-based models. These are critical for understanding the contributions of specific genes and pathways to DS and for evaluating potential pharmacological interventions.

Animal models inherently contain mitigations to these various concerns. For tissue complexity, animal models present a self-contained multi-organ system, with accurate cellular, mechanical, and systemic interaction. This combined with well-characterized developmental time-courses make aberrations, such as congenital organ scale defects, easily apparent. Further, behavior can be easily studied in animal models, allowing for connections to genetics. Thus, cellular and mouse models are complementary, and an understanding of DS may be improved by using them together.

### Translational and clinical perspectives

Fundamental or basic research begins with questions about mechanisms underlying outcomes. In the case of trisomy 21, perhaps the most basic observation is that an increase in gene template availability results in changes – primarily up regulation but in some cases little effect or down regulation – of steady state transcript levels.^187^^,^^188^ Resulting changes to protein concentration will have an immediate effect on transcripts of genes on other chromosomes, for example when the 7 transcription factors on Hsa21 are over-expressed. A critical but difficult-to-measure perturbation of altered timing, as critical signaling molecules or increased receptors reach activation thresholds for developmental pathways earlier (or later). These disruptions then have a cascading effect on development.^189^^,^^190^ Ultimately the balance that is reached will be slightly altered in molecular terms, while the derived equilibrium will be subtly but significantly altered to produce features of DS. It should be noted that an estimated 50% of conceptuses with trisomy 21 do not survive to term.^191^

This deceptively simple gene-dosage framework has progressively evolved toward a systems-level understanding of trisomy 21 as a disorder of regulatory imbalance rather than linear overexpression. Increasing evidence indicates that trisomy induces genome-wide transcriptional remodeling, chromatin reorganization, and altered epigenetic landscapes that extend far beyond Hsa21 itself.^192^^,^^193^ Thus, phenotypes most likely do not arise from isolated 1.5-fold increases in protein abundance, but from network-level shifts in transcriptional thresholds, developmental timing, and signaling responsiveness.

The search for a “Master Down Syndrome Gene”, normalization of which would eliminate all effects of trisomy, has been largely replaced by “genes of major effect” for a given phenotypic manifestation of trisomy 21.^194^ An extra copy of *Ets2* has been shown in mouse models to provide most of the resistance to familial adenomatous polyposis (colon cancer) arising from identical mutations in the human and mouse *ApcMin* genes.^195^ *SOD1*, one of the first genes mapped to Hsa21 and the most studied for a number of years, was asserted to “cause” many DS features when over-expressed in transgenic mice, although much of the early work on this gene may have been influenced by the use of an artificial promoter changing timing, location and levels of expression.^196^ Toxic gain-of-function mutations in this gene contribute substantially to the development of amyotrophic lateral sclerosis (ALS), a condition with no obvious close association to features of DS. Overexpression of Synaptojanin 1 (*SYNJ1*) has been strongly associated with multiple phenotypes related to synaptic transmission and with altered processing of another major Hsa21 gene, *APP*. These effects are thought to arise from SYNJ1’s critical role in the endocytic machinery.^197^^,^^198^^,^^199^ Lastly, and importantly, overexpression of *APP* is the primary driver of AD in individuals with DS and is widely regarded as the major gene underlying this comorbidity.^166^^,^^200^^,^^201^

Among genes of major effect, *DYRK1A* has emerged as one of the most compelling translational nodes. *Dyrk1a* dosage has been associated with a snowballing number of DS features with varying degrees of evidence supporting or refuting these roles. The overexpression of this gene contributes to altered neurogenesis, impaired synaptic plasticity, dendritic abnormalities, and cognitive dysfunction in trisomic mouse models.^202^^,^^203^^,^^204^ DYRK1A also modulates tau phosphorylation and intersects mechanistically with AD pathways, which are universally accelerated in individuals with trisomy 21.^205^ Importantly, pharmacological inhibition of DYRK1A has been shown to ameliorate cognitive and synaptic deficits in mouse models, underscoring its druggability and translational relevance.^206^ Inhibitors like Leucettine L41 have shown normalization of Dyrk1a activity, correction of certain cognitive impairments, and remodeling of the underlying functional connectivity of core brain areas in mouse models of DS.^207^ Combinatorial strategies such as the administration of EGCG (epigallocatechin-3-Gallate), another Dyrk1a inhibitor, along with environmental enrichment in Young Ts65Dn mice, improved corticohippocampal learning and memory more than either treatment alone.^208^^,^^209^^,^^210^ Attenuation of hippocampal synaptic plasticity was also seen by normalization of Dyrk1a levels using AAV mediated transduction of inhibitory short hairpin RNA in Ts65Dn.^211^ These effects show that rather than representing a “master gene”, *DYRK1A* may function as a dosage-sensitive kinase hub whose normalization can partially recalibrate downstream networks. Consequently, work on this dosage correction translated into clinical trials of promising DYRK1A inhibitors.^206^^,^^212^

Beyond coding genes, trisomy 21 also perturbs non-coding RNA networks and epigenetic regulators.^193^ Modulation of miRNA dosage through sponge-based strategies can normalize specific dysregulated targets in DS cellular and mouse models, illustrating that dosage-sensitive genes such as *DYRK1A* can be indirectly rebalanced through post-transcriptional intervention.^203^ Recent studies have further explored long non-coding RNAs such as *SNHG11* and other regulatory RNAs as modulators of neuronal differentiation and synaptic homeostasis, reinforcing the view that DS involves altered post-transcriptional and chromatin-level regulation rather than simple gene excess.^213^

Genome-wide DNA methylation and chromatin accessibility studies reveal consistent epigenomic remodeling in trisomy 21, supporting the concept that DS may be understood as a chromatin and transcriptional timing disorder.^192^^,^^193^ These epigenetic shifts are inherently dynamic and, importantly, pharmacologically modifiable. Histone deacetylase inhibitors, kinase inhibitors targeting DYRK1A, and emerging epigenetic editing tools represent potential strategies to normalize dysregulated transcriptional states.

An additional innovative translational avenue in rodent models explored the role of retrotransposon activity in neurodegeneration. Increased LINE-1 activity and transposable element dysregulation have been implicated in aging and AD. Repurposing reverse transcriptase inhibitors such as lamivudine has shown promise in modulating retrotransposon-associated pathology and neuroinflammatory responses in experimental systems.^214^^,^^215^

Evidence from rare partial trisomy cases lacking *APP* triplication, together with familial AD cases involving duplication of *APP*-containing chromosomal regions, demonstrates that increased *APP* gene dose is a key driver of AD pathogenesis in DS.^216^^,^^217^^,^^218^^,^^219^ Established DS mouse models have confirmed the contribution of excess APP to multiple AD-related pathologies and provide platforms for identifying and testing druggable targets.^166^^,^^200^^,^^220^^,^^221^^,^^222^ Genetic normalization of *App* copy number in Ts65Dn and Dp(16)1Yey mice defined its central role. Elevated APP promotes increased Aβ production, tau hyperphosphorylation, impaired neurotrophin transport and signaling, retromer dysfunction, synaptic protein loss, and neuroinflammation.^166^^,^^200^^,^^220^^,^^221^^,^^222^ These findings support therapeutic strategies aimed at lowering APP expression or modifying its processing.

Pharmacological approaches using Posiphen and APP-specific antisense oligonucleotides (ASOs) reduced APP expression to euploid levels in Ts65Dn and Dp(16)1Yey DS mouse models, respectively, decreased toxic APP fragments (β-CTF and Aβ42), and reversed multiple AD-associated phenotypes.^222^^,^^223^ These results highlight strong translational potential. Posiphen has advanced to Phase III trials for early-onset AD, and APP-ASO is being planned for clinical testing in DS. Recent studies further identify Rab5 hyperactivation, downstream of APP overexpression, as a central regulator of AD pathology. RAB5-targeting ASOs rescued several AD-related phenotypes in Dp(16)1Yey mice, suggesting that both APP and Rab5 are promising therapeutic targets.^223^ γ-secretase modulators (GSMs) were developed to selectively regulate the enzyme’s activity by enhancing γ-secretase processivity, thus reducing longer and more aggregation-prone Aβ42 and Aβ40 peptides.^224^^,^^225^ Novel GSMs with improved potency and brain penetration—such as BPN-15606 and UCSD-776890—have shown strong preclinical efficacy.^226^^,^^227^^,^^228^^,^^229^ In Ts65Dn mice, BPN-15606 reduced toxic Aβ42 and Aβ40 without altering total APP and ameliorated AD-related pathology.^229^

Additionally, anti-amyloid monoclonal antibodies, including Aducanumab, Lecanemab, and Donanemab, have been approved for AD, reinforcing the amyloid hypothesis.^230^ However, individuals with DS have largely been excluded from these trials, except for an ongoing Donanemab study in non-demented adults with DS. Vaccine-based anti-Aβ approaches in Ts65Dn mice have similarly reduced brain Aβ, improved memory, and decreased cholinergic neuron loss.^231^ The Phase 1b/2 ABATE trial is also evaluating ACI-24.060 in adults with DS at risk for AD, with interim results indicating acceptable safety.

The evaluation of underlying circuit changes resulting in atypical behavior has birthed a now well-established hypothesis of overinhibition and an overall excitatory-inhibitory imbalance in the brain.^232^^,^^233^^,^^234^^,^^235^ Targeting this, treatments such as chronic fluoxetine treatment in adult Ts65Dn mice have been shown to normalize GABA release and restore hippocampal synaptic plasticity, leading to improvements in spatial memory and working memory.^236^ Targeting GABA-receptor mediated over-inhibition, short-term oral administration of Pentylenetetrazol (PTZ), a GABAA receptor antagonist, improved long-term recognition memory and decision-making but not short-term memory.^237^^,^^238^ Additionally, chronic treatment of Ts65Dn animals with a selective GABAA α5 negative allosteric modulator (NAM) rescued functional and structural brain parameters, such as improving hippocampal plasticity, neurogenesis, and restoring cognitive function.^239^ Restorative effects on neural proliferation and cognition have also been observed following prenatal treatment with natural product-derived therapeutics such as Oleic Acid or Linolenic Acid, Curcumin, and EGCG.^209^^,^^210^^,^^240^^,^^241^^,^^242^ Another development-based treatment - Maternal choline supplementation - has shown enhanced hippocampal function and learning in Ts65Dn.^243^^,^^244^^,^^245^ It has also shown to be effective in prevention of age-associated neurodegeneration of cholinergic and GABAergic basal forebrain neurons in Ts65Dn.^246^ Given the limitations of the Ts65Dn model in terms of construct validity, future studies could focus on testing promising therapeutic candidates on more recent models of DS which have improved construct validity. Furthermore, a related collective effort could include the integration of results from clinical and preclinical studies to enable the development of future therapeutic strategies.^247^ This continuous iterative process encompassing both forward and reverse translation has significant potential to develop efficacious treatments for a host of conditions experienced by persons with DS. Another promising path forward could also make use of powerful large language models (LLM) and agentic architectures to mine novel targets and mechanisms from the growing biomedical DS corpus.^248^^,^^249^^,^^250^

### Prioritization of research efforts

Now that future-facing translational efforts have been covered, an important question for researchers remains: which features of DS should be prioritized for research? For fundamental level understanding, the goal of which is to gain new knowledge of things currently unknown, it would be difficult if not antithetical to greatly restrict inquiry. However, the discovery path to translation is a long way from initial observations, and the approaches and techniques are modeled on the desired goals. For example, studies of midfacial skeletal retrusion that contributes substantially to facial appearance, restricted sinus passages, dentition and jaw issues would likely have a different focus than those trying to ameliorate genetic effects contributing to the high frequency of structural heart defects (except generally, for NCC contributions). Examination of compounds that might affect neurotransmission to facilitate learning and memory would require a different set of studies. Given the different categories of possible investigation, how should self-advocate and caregiver priorities be determined and factored into a system to optimize efforts that allow individuals with trisomy 21 to reach their full potential? This ongoing conversation between those directly affected, those attempting to develop interventions, and the health care delivery needed to effect solutions might profitably be bolstered. A helpful path forward for translational prioritization should integrate biological tractability, developmental timing, clinical burden, and stakeholder priorities. Cognitive dysfunction, AD risk, immune dysregulation, and metabolic vulnerability represent partially overlapping but mechanistically distinct domains. A network-based and epigenetically informed framework would allow for targeted interventions that may shift biological trajectories without requiring chromosomal correction. It is helpful to keep in mind that despite facing real health challenges, persons with DS have significantly positive self-perception and contentment.^251^ Considering this, a valuable objective for preclinical DS research would be to identify mechanisms to enhance resilience across the lifespan, with the ultimate goal of enabling persons with DS to live fuller and freer lives.

## Conclusion

This primer is a valuable resource since most if not all the research teams that have generated and characterized both the established as well as the novel and most advanced rodent models of DS are represented among the authors of this manuscript. The complexity of modeling DS in mice and rats remains a fundamental challenge. However, there is now a wide range of rodent models to help investigations into the dosage sensitive genes on Hsa21 that cause phenotypes, and the mechanisms by which they do so. If a particular phenotype is of interest, an investigator can avail themselves of the many genetically well-defined rodent models, including the ‘zoo’ of mice with different regions of triplication containing different Hsa21 homologous genes. This will allow the investigator to pinpoint candidate regions and even candidate genes for investigating causality. An alternative approach would be to start from a well-defined animal model and investigate phenotype in a way that may not be possible with humans – this approach often leads to new insights as it did with insights into cerebellar development in DS. In all efforts using rodent models it is essential that the animals are used ethically and with respect, minimizing numbers in carefully designed experiments. It is also essential that the most appropriate model is used, with understanding of its advantages and limitations. Considering the differences between models, the reader can make a prudential decision eschewing questions along the lines of which model is “the best”. Rather, given the detailed descriptions of genomic capture and model-specific phenotypes surveyed in this primer, a more helpful approach would be to think first about the scientific question at hand and then think about which model is “the best for” addressing that particular question. Along the same lines, it is also recommended that readers use more than one model of DS to gain a comprehensive understanding of pathophysiological mechanisms rather than rely on a single model to draw concrete conclusions. Economic factors are also worthwhile considerations for readers since some mouse models of DS have issues with breeding, fertility, and reproductive success. Resources such as the cytogenetics and DS resource funded by the National Institutes of Health (NIH) in the United States that provides subsidized costs for NIH-funded researchers is a step in the right direction to help offset costs for maintaining animal colonies. As has been made clear in this comprehensive guide, rodent models will continue to remain the bedrock of understanding DS, hand in hand with other preclinical models and clinical approaches.

## Acknowledgments

We gratefully acknowledge the funding agencies and institutions that supported this work. A.S. was supported by the 10.13039/501100001673Jérôme Lejeune Foundation Advanced Pilot Grant (project no. 2008) and a T21RS Early Investigators Award (2023). M.A.R. was supported by the University of Dayton Graduate Student Summer Fellowship and the Bro. Don Geiger Graduate Award. Y.E.Y. was supported by the Children's Guild Foundation and the 10.13039/100000002National Institutes of Health (R01HD109750 and R01DC019735). A.F. and R.J.R. were supported by the 10.13039/100000002NIH (HD118475). We acknowledge 10.13039/100000002NIH F32HL178155 to L.K.B. We acknowledge 10.13039/100000957Alzheimer's Association Grant AARG-22-973859 to A.T. M.C.F. and V.L.J.T. were supported by the 10.13039/100010269Wellcome Trust (grant nos. 080174, 098327, and 098328). V.L.J.T. was also supported by the 10.13039/100010438Francis Crick Institute (CC2080), which receives its core funding from 10.13039/501100000289Cancer Research UK(CC2080), the 10.13039/501100000265UK Medical Research Council (CC2080), and the 10.13039/100010269Wellcome Trust (CC2080). K.W. was supported by the 10.13039/501100001673Jérôme Lejeune Foundation (project no. 1920). M.D. received funding from the 10.13039/501100011033Agencia Estatal de Investigación (PID2022-141900OB-I00 funded by MICIU/10.13039/100005910AEI/10.13039/501100011033/10.13039/501100008530ERDF, EU), 10.13039/100008666Fundació La Marató-TV3 (#202212-30), 10.13039/100014440Ministerio de Ciencia, Innovación y Universidades (RTC2019-007230-1 and RTC2019-007329-1; CPP2022-009659 funded by 10.13039/501100004837MCIN/AEI/10.13039/501100011033 and EU’s 10.13039/100031478NextGenerationEU/PRTR). The CIBER of Rare Diseases is an initiative of the ISCIII. The lab of M.D. is recognized by the Secretaria d’Universitats i Recerca del Departament d’Economia I Coneixement de la Generalitat de Catalunya (Grups consolidats 2023). We further acknowledge the support of the 10.13039/501100004837Spanish Ministry of Science and Innovation through the Centro de Excelencia Severo Ochoa (CEX2020-001049-S, 10.13039/501100004837MCIN/AEI/10.13039/501100011033), the 10.13039/501100010552Generalitat de Catalunya through the 10.13039/100015439CERCA program, and the 10.13039/100013060EMBL partnership. We acknowledge the 10.13039/100009619Japan Agency for Medical Research and Development (AMED) under grant no. JP26ama121046 to Y.K.; Y.H. was supported in part by the Interdisciplinary Thematic Institute IMCBio+, as part of the ITI 2021–2028 program of the 10.13039/501100003768University of Strasbourg, 10.13039/100012681CNRS, and 10.13039/501100001677Inserm; by IdEx Unistra (ANR-10-IDEX-0002), the SFRI-STRAT'US project (ANR-20-SFRI-0012), EUR IMCBio (ANR-17-EURE-0023), and INBS PHENOMIN (ANR-10-IDEX-0002-02) under the France 2030 Program; by the 10.13039/100007353Fondation Jérôme Lejeune; and by the 10.13039/501100001665Agence Nationale de la Recherche through the projects DendriDown (ANR-22-CE16-0021), Transbioroyal (ANR-22-CE18-0031), DevInDS (ANR-21-NEU2-0011), and GENISIF-DS21 (ANR-25-CE16-3065). Additional support to Y.H. was provided by the EU-funded project GO-DS21 (grant agreement ID: 848077).

## Author contributions

Conceptualization: M.A.R. and A.S.; visualization: M.A.R., Z.X., Y.E.Y., and A.S.; writing – original draft: all authors; writing – review and editing: all authors.

## Declaration of interests

R.J.R. is a member of the executive board of the Trisomy 21 Research Society.
